# Development of a biomimetic thyroid acellular scaffold as a 3D platform for modeling thyroid cancer aggressiveness and drug resistance

**DOI:** 10.3389/fbioe.2025.1692549

**Published:** 2025-11-12

**Authors:** Liang Zhang, Rong Sun, Shuheng Li, Peng Zhang, Houlong Long, Bin Liu, Feng Li

**Affiliations:** 1 Department of Thyroid and Breast Surgery, Tengzhou Central People’s Hospital Affiliated to Xuzhou Medical University, Tengzhou, China; 2 Department of Thyroid and Breast Surgery, Tengzhou Central People’s Hospital, Tengzhou, China

**Keywords:** acellular scaffold, thyroid cancer, 3D tumor model, tumor microenvironment, drugresistance, BRAF V600E, epithelial-mesenchymal transition

## Abstract

Traditional two-dimensional (2D) cell culture models for thyroid cancer research fail to recapitulate the complex tumor microenvironment (TME), leading to a significant gap between preclinical findings and clinical outcomes. To address this limitation, this study focuses on the development and characterization of a novel three-dimensional (3D) tumor model utilizing a thyroid acellular scaffold (TAS) derived from rat tissue. We prepared the TAS through an optimized decellularization protocol, followed by comprehensive histological, biochemical, proteomic, and mechanical evaluations. Human thyroid cancer cells were then seeded onto the TAS, and their biological behaviors, including proliferation, invasion, gene expression, and drug sensitivity to cisplatin and vemurafenib, were systematically compared to conventional 2D cultures. Our results demonstrate that the TAS provides a biomimetic microenvironment, successfully preserving the native extracellular matrix (ECM) architecture, key proteins, and a significant fraction of endogenous growth factors. Compared to 2D cultures, cells within the 3D TAS model exhibited significantly enhanced proliferation and time-dependent invasion. Critically, the 3D microenvironment induced a more aggressive phenotype, characterized by upregulated expression of the BRAF V600E oncogene and the induction of epithelial-mesenchymal transition (EMT), and conferred significantly increased resistance to both cisplatin and vemurafenib. These findings indicate that our tissue-specific, TAS-based 3D model successfully recapitulates key pathophysiological hallmarks of thyroid cancer, representing a more clinically relevant and predictive platform for investigating tumor mechanisms and for the preclinical evaluation of novel therapeutic agents.

## 1 Introduction

Thyroid cancer is the most rapidly increasing endocrine malignancy worldwide, particularly among younger populations, posing a growing challenge to public health (Pizzato et al., 2022). According to the latest global cancer statistics, its incidence has continued to rise over the past few decades, constituting a major endocrine cancer burden (Sung et al., 2021). Differentiated thyroid cancer (DTC) is the predominant pathological type, accounting for approximately 95% of all cases. Although most DTC patients have a favorable prognosis, tumor recurrence, metastasis, and resistance to conventional therapies such as radioactive iodine (RAI) and chemotherapy remain key factors leading to treatment failure and mortality (Filetti et al., 2019). In particular, radioactive iodine-refractory (RAIR) thyroid cancer has limited treatment options and an extremely poor prognosis (Veschi et al., 2020). Therefore, a deeper understanding of the mechanisms of thyroid cancer progression and the development of more effective therapeutic strategies are urgent issues in current clinical and basic research.

Currently, cancer biology research and preclinical drug screening largely rely on traditional two-dimensional (2D) monolayer cell culture models. However, these models, where cells grow on flat, rigid plastic surfaces, greatly oversimplify the *in vivo* tumor environment. They completely neglect the complex three-dimensional (3D) topology, the dynamic and intimate interactions between cells and the extracellular matrix (ECM), and the rich biochemical signals and physical-mechanical cues within the tumor microenvironment (TME) (Kothari et al., 2016; Mao et al., 2021). This fundamental flaw in the model prevents it from accurately mimicking the true proliferation patterns, invasive potential, gene expression profiles, and drug responses of tumor cells *in vivo* (Langhans, 2018). This is considered one of the key reasons for the significant gap between preclinical research findings and clinical trial outcomes, known as the “preclinical-to-clinical” translation failure (Ishiguro et al., 2017). Consequently, developing 3D *in vitro* models that can highly mimic the *in vivo* tumor microenvironment has become a critical challenge in the field of oncology research (Jensen and Teng, 2020).

This need for more predictive *in vitro* systems is a challenge shared across oncology. In the analogous field of Head and Neck Squamous Cell Carcinoma (HNSCC), for example, it is well-defined that commercial cell lines on 2D supports fail to recapitulate the TME. Consequently, a strong emphasis has been placed on developing 3D preclinical models. These advanced systems, including patient-derived organoids, organotypic co-cultures, and collagen-based scaffolds, have been shown to be valuable resources that more faithfully reproduce the biological behaviors of HNC tumors and microenvironmental interactions (Engelmann et al., 2020; Millen et al., 2023; Miserocchi et al., 2021). The success of these models in HNSCC further underscores the critical importance of shifting from 2D to 3D platforms, a principle that this study now applies to the field of thyroid cancer.

Tissue engineering techniques, particularly the use of decellularized extracellular matrix (dECM) as a scaffold, offer a promising strategy for constructing high-fidelity 3D tumor models (Rodrigues et al., 2022). Compared to synthetic polymers (e.g., polyethylene glycol) or single-component natural polymers (e.g., collagen, gelatin) (Horder et al., 2021), dECM scaffolds derived from specific organs possess unparalleled advantages. They can maximally preserve the unique ECM components of the original tissue (such as different types of collagen, laminin, fibronectin), the nano-scale fibrous network topology, and the sequestered endogenous growth factor profile (Choi et al., 2023; Karamanos et al., 2021). This “tissue specificity” is crucial for simulating the microenvironment of a particular cancer, as it provides tumor cells with a “home” that is highly similar to their *in vivo* niche (Hinderer et al., 2016; Crouigneau et al., 2024).

However, a comprehensively characterized 3D *in vitro* model derived from thyroid tissue for thyroid cancer research is still lacking. This study aims to prepare and systematically characterize a thyroid acellular scaffold (TAS) derived from rat thyroid tissue through an optimized decellularization method, and to establish a novel human thyroid cancer 3D *in vitro* model based on it. We hypothesize that, compared to traditional 2D culture models, this tissue-specific scaffold-based 3D model can more realistically recapitulate the biological behaviors of thyroid cancer and reveal the critical role of the microenvironment in regulating tumor cell proliferation, invasion, and drug sensitivity.

**FIGURE 1:**
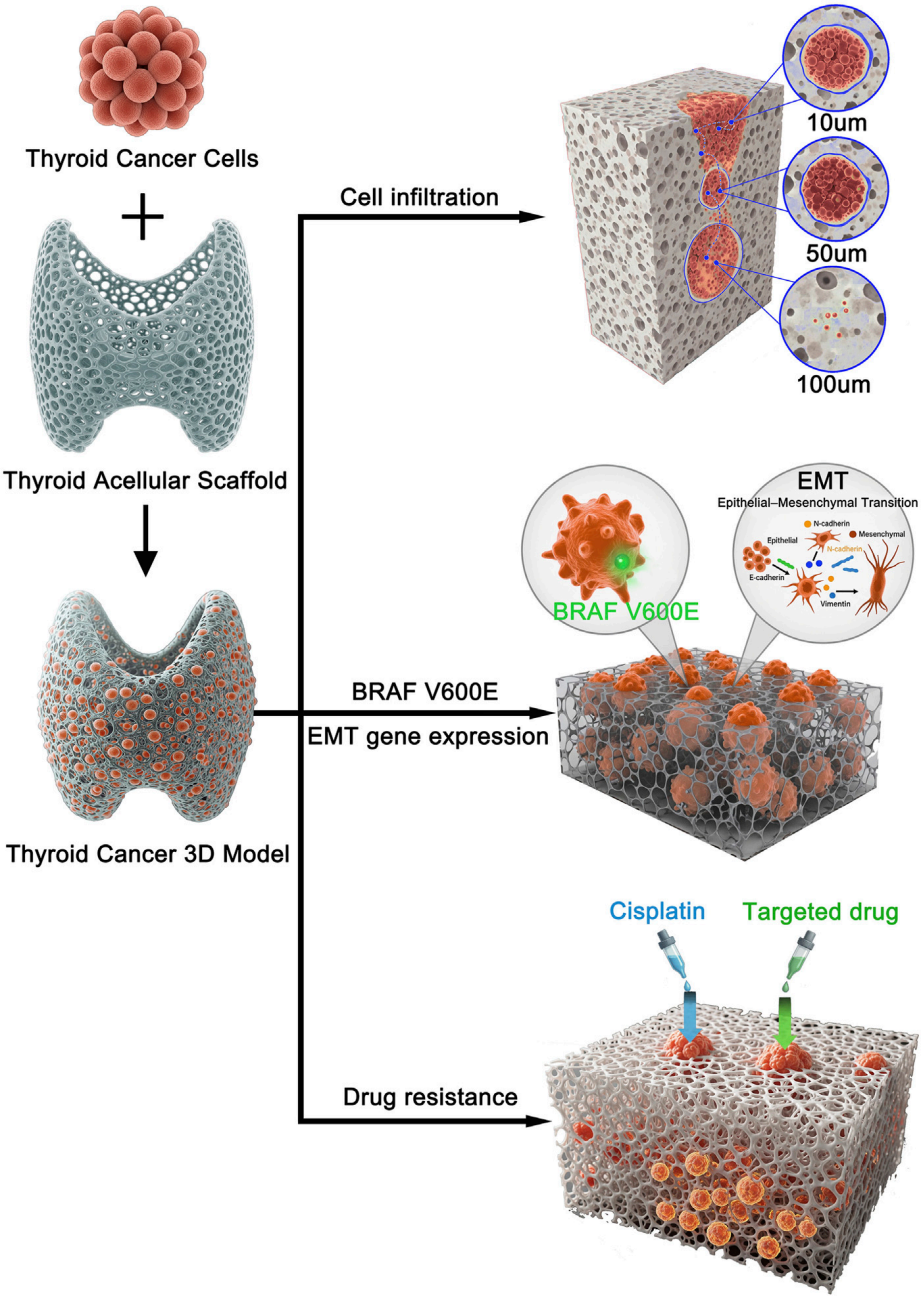
Schematic illustration of the experimental design and key findings for the 3D thyroid cancer model. Human thyroid cancer cells were seeded onto a prepared thyroid acellular scaffold to construct a 3D *in vitro* tumor model. This biomimetic model was subsequently used to investigate key cancer progression and treatment-related characteristics. The study revealed three main findings: (1) Cancer cells successfully infiltrated the scaffold, with progressive invasion observed at depths of 10, 50, and 100 µm over time. (2) The 3D microenvironment promoted cancer progression by upregulating BRAF V600E expression and inducing an epithelial-mesenchymal transition (EMT) phenotype. (3) The 3D model exhibited significantly increased resistance to both conventional chemotherapy (Cisplatin) and a targeted drug (Vemurafenib) compared to 2D cultures.

To test this hypothesis, we first conducted a comprehensive characterization of the prepared TAS using histological, biochemical, proteomic, and mechanical testing methods to confirm the effective removal of cellular components and the preservation of key ECM components, growth factors, and mechanical properties. Subsequently, we seeded human thyroid cancer cells (BCPAP) onto the TAS to construct a 3D tumor model and compared them with cells cultured in 2D. We systematically evaluated the differences between the two models in terms of cell proliferation kinetics, invasion and penetration into the scaffold, and the expression of genes related to epithelial-mesenchymal transition (EMT). Finally, we assessed and compared the sensitivity of the two models to the commonly used chemotherapy drug cisplatin and the BRAF V600E targeted drug vemurafenib (Figure 1). The high-fidelity 3D model established in this study is intended to provide a more reliable and clinically predictive preclinical research platform for exploring the pathogenesis of thyroid cancer and developing more effective therapeutic strategies.

## 2 Materials and methods

### 2.1 Preparation of thyroid acellular scaffold (TAS)

#### 2.1.1 Experimental animals and thyroid tissue harvesting

Healthy adult Wistar rats (weighing approximately 250–300 g) were purchased from the Experimental Animal Center of Xuzhou Medical University. All animal experiments were conducted in accordance with the guidelines of and were approved by the Animal Ethics Committee of Xuzhou Medical University. Rats were anesthetized via intraperitoneal injection of sodium pentobarbital (40 mg/kg) to ensure a painless procedure. On a sterile operating table, the neck region of the rat was carefully dissected to expose, identify, and isolate the thyroid gland. Surrounding tissues were covered with saline-moistened gauze to prevent drying and damage. The thyroid gland was carefully separated from adjacent tissues using anatomical scissors and forceps to ensure its integrity. The harvested thyroid tissue was immediately placed in pre-chilled phosphate-buffered saline (PBS) to remove blood and adipose tissue.

#### 2.1.2 Decellularization protocol

The thyroid tissue blocks were placed in deionized water and agitated at 4 °C for 24 h to remove soluble extracellular proteins and carbohydrates. The tissues were then rinsed three times with deionized water for 5 min each. The tissue blocks were subsequently immersed in a 0.5% sodium dodecyl sulfate (SDS, Sigma-Aldrich, L3771) solution and agitated at room temperature for 24 h. After treatment, the tissues were rinsed three times with deionized water for 5 min each. Next, the tissue blocks were placed in a 1% Triton X-100 (Sigma-Aldrich, T8787) solution and agitated at room temperature for 24 h. Following this step, the tissues were again rinsed three times with deionized water for 5 min each. Finally, to ensure the complete removal of detergent residues, the scaffolds were thoroughly rinsed in deionized water for 72 h with frequent changes (every 6 h). The conductivity and UV absorbance (at 280 nm) of the rinsing solution were monitored until they matched those of ultrapure water, indicating the effective elimination of SDS and Triton X-100. The purified thyroid acellular scaffolds were then freeze-dried, ground into a fine powder in liquid nitrogen, and stored at −80 °C for future use.

#### 2.1.3 Standardization and reproducibility assurance

To ensure batch-to-batch reproducibility of the Thyroid Acellular Scaffold (TAS), a rigorous quality control strategy was implemented. This began by sourcing thyroid tissue exclusively from a homogenous population of adult Wistar rats (250–300 g) to minimize biological variability. A strictly standardized decellularization protocol was then applied to all tissues. For experimental consistency, all resulting scaffolds were trimmed to uniform dimensions (5 mm × 5 mm × 3 mm). Finally, comprehensive characterization of at least three independent batches via histology, SEM, and mechanical testing confirmed low inter-batch variability in decellularization efficacy, ECM architecture, porosity, and mechanical properties.

### 2.2 Characterization of the thyroid acellular scaffold

#### 2.2.1 Assessment of decellularization efficacy

The efficiency of thyroid decellularization was systematically evaluated by measuring the content of DNA, sulfated glycosaminoglycans (sGAG), and collagen before and after decellularization. DNA Content Assay: 100 mg of thyroid tissue before and after decellularization was freeze-ground into powder. DNA was extracted and quantified using the PicoGreen DNA Quantitation Kit (Invitrogen, P7589). Fluorescence intensity was measured using a fluorescence microplate reader (SpectraMax i3x, Molecular Devices) at an excitation wavelength of 480 nm and an emission wavelength of 520 nm to calculate the DNA content. sGAG Content Assay: Tissues were digested with papain (Sigma-Aldrich, P4762) in a 60 °C water bath for 24 h. The sGAG content was then determined using the dimethylmethylene blue (DMMB) assay. The sGAG content before and after decellularization was calculated and compared based on a standard curve. Collagen Content Assay: Collagen was extracted and quantified using the Sircol Collagen Assay Kit (Biocolor, S1000). Absorbance was measured at 540 nm using a fluorescence microplate reader, and the content was calculated based on a standard curve.

#### 2.2.2 Histological and morphological analysis

H&E and Masson’s Trichrome Staining: Thyroid tissues before and after decellularization were sectioned into 5 μm thick slices, fixed with 4% paraformaldehyde for 15 min, and then subjected to H&E and Masson’s trichrome staining. An optical microscope (Leica DM 2000, Leica Microsystems) was used to observe the tissue structure, the removal of cell nuclei, and the distribution and preservation of collagen fibers. DAPI Staining: To further confirm the removal of cell nuclei, tissue sections before and after decellularization were fixed with 4% paraformaldehyde for 10 min. They were then stained with DAPI solution (1:1,000, Thermo Fisher Scientific) for 5 min to visualize any residual nuclei. A fluorescence microscope (Leica DM 2000) was used to observe and capture images to confirm the complete removal of nuclei. Scanning Electron Microscopy (SEM): Tissue blocks before and after decellularization were cut into approximately 1–2 mm^3^ pieces, fixed with 2.5% glutaraldehyde at 4 °C for 2 h, and post-fixed with 1% osmium tetroxide for 1 h. After graded ethanol dehydration, critical point drying, and gold sputtering, a scanning electron microscope (Hitachi, S-4800) was used to observe the cellular structure, ECM preservation, and the integrity of the fibrous network.

#### 2.2.3 Preservation of major extracellular matrix proteins

Immunofluorescence staining was used to assess the retention of key proteins, including Collagen I (Abcam, ab270993), Collagen IV (Abcam, ab6586), Fibronectin (Abcam, ab268020), and Laminin (Abcam, ab108536). Tissue sections were fixed, permeabilized, and blocked, followed by overnight incubation with primary antibodies at 4 °C. After washing, a fluorescent secondary antibody (Alexa Fluor 594, Abcam, ab150080) was added and incubated for 1 h at room temperature. Nuclei were counterstained with DAPI. A fluorescence microscope (Leica, DM 2000) was used to observe and compare staining intensity and distribution.

#### 2.2.4 Assessment of residual detergents and cytotoxicity of TAS extracts

To confirm the biosafety of the decellularized scaffold, TAS extracts were prepared following the ISO 10993-5 standard. Briefly, 0.2 g of sterilized TAS samples were immersed in 1 mL of serum-free DMEM and incubated at 37 °C for 24 h. The supernatant was collected and filtered to obtain the extraction medium. BCPAP cells were seeded into 96-well plates (1 × 10^4^ cells/well) and incubated with TAS extracts or control medium for 48 h. Cell viability was quantified using the CCK-8 assay. The viability of cells exposed to TAS extracts exceeded 95% compared with the control group, demonstrating negligible cytotoxicity. These results confirmed that residual SDS and Triton X-100 were effectively removed during the washing process.

### 2.3 Proteomic analysis of the thyroid acellular scaffold

To evaluate protein retention, mass spectrometry was employed. 100 mg each of thyroid tissue before and after decellularization was freeze-ground into powder. The powder was dissolved in lysis buffer (Sigma-Aldrich), sonicated (Branson Ultrasonics), and centrifuged (Eppendorf, Centrifuge 5417R) to collect the supernatant for total protein extraction. Protein concentration was determined using the BCA Protein Assay Kit (Thermo Fisher Scientific). Samples were incubated with 10 mM dithiothreitol (DTT, Sigma-Aldrich) at 37 °C for 1 h, then alkylated with 55 mM iodoacetamide (IAA, Sigma-Aldrich) in the dark for 30 min. Urea was diluted to 2 M, and trypsin (Promega, 1:50, w/w) was added for overnight digestion at 37 °C. The reaction was terminated and acidified with 0.1% formic acid (Thermo Fisher Scientific). Peptides were analyzed by liquid chromatography-tandem mass spectrometry (LC-MS/MS) using a NanoLC system (Thermo Fisher Scientific) and a mass spectrometer (Thermo Fisher Scientific, Q-Exactive HF). A data-dependent acquisition (DDA) mode was used, selecting the top 20 most intense signals for fragmentation. Data analysis was performed using Proteome Discoverer software (Thermo Fisher Scientific), with peptide and protein identification and quantification conducted using a label-free quantification (LFQ) method. Protein abundance was calculated using intensity-based absolute quantification (iBAQ). The protein abundance in the decellularized scaffold was compared to that before decellularization to assess the retention of key proteins. Changes in protein abundance were expressed as log2 ratios (post-decellularization/pre-decellularization) to reveal the retention or loss trends of different proteins during the decellularization process.

### 2.4 Growth factor content assay of the thyroid acellular scaffold

Enzyme-linked immunosorbent assay (ELISA) was used to quantify the content of key growth factors in the scaffold. Samples of native thyroid tissue and decellularized scaffold were cut into small pieces and placed in lysis buffer containing protease inhibitors for growth factor extraction. The samples were sonicated, centrifuged, and the supernatant was collected. Specific ELISA kits were used to detect the content of vascular endothelial growth factor (VEGF, R&D Systems, RRV00), transforming growth factor-β (TGF-β, R&D Systems, RTB100B), hepatocyte growth factor (HGF, R&D Systems, MHG00), fibroblast growth factor (FGF, R&D Systems, MFB00), epidermal growth factor (EGF, R&D Systems, DY3214), and platelet-derived growth factor (PDGF, R&D Systems, MBB00). The procedure was performed according to the kit instructions, and absorbance was measured at 450 nm using a microplate reader. The concentration of each growth factor (pg/mL) was calculated from a standard curve. Each sample was tested in triplicate, and at least three independent experiments were performed.

### 2.5 Mechanical property testing of the thyroid acellular scaffold

The tensile properties of native rat thyroid and the thyroid acellular scaffold were evaluated using a TRAPEZIUM materials testing machine (Shimadzu, Japan). Test samples (15 mm × 10 mm × 2 mm) were cut from native rat thyroid tissue and the decellularized scaffold. Before testing, samples were immersed in PBS at room temperature for 1 h to simulate physiological conditions and maintain hydration. During the test, samples were fixed in the clamps of the materials testing machine with a gauge length set to 10 mm to standardize the test length and ensure uniform stress distribution during stretching. Uniaxial tensile testing was performed at a constant strain rate of 2 mm/min until the sample ruptured. The stress-strain curve was recorded in real-time. The maximum elastic modulus was calculated from the slope of the steepest part of the curve, and the maximum stress before rupture (defined as the ultimate tensile strength, UTS) was also recorded.

### 2.6 Construction and culture of the 3D in vitro tumor model

#### 2.6.1 Cell culture

The human thyroid cancer cell line BCPAP (ATCC) was used in this study. Cells were cultured in Dulbecco’s Modified Eagle Medium (DMEM, Gibco) supplemented with 10% fetal bovine serum (FBS, Gibco) and 1% penicillin-streptomycin solution (Gibco). The culture environment was maintained at 37 °C with 5% CO2 in a humidified incubator (Thermo Fisher Scientific). Cryopreserved cells were rapidly thawed in a 37 °C water bath. After thawing, cells were centrifuged to remove dimethyl sulfoxide (DMSO, Sigma-Aldrich) and resuspended in fresh complete medium. The cell suspension was transferred to a culture flask and incubated. When cells reached 80%–90% confluence, they were digested with 0.25% trypsin-EDTA solution (Gibco) for subculturing.

#### 2.6.2 Construction and culture of the 3D tumor model

The thyroid acellular scaffold was trimmed into 5 mm × 5 mm × 3 mm blocks and hydrated in sterile PBS for 2 h to restore its softness. The hydrated scaffolds were then immersed in PBS containing 100 U/mL penicillin and 100 μg/mL streptomycin and gently agitated at 4 °C for 12 h for thorough sterilization. The sterilized scaffolds were rinsed 3 times with sterile PBS to remove residual antibiotics.

A cell suspension of human thyroid cancer cells BCPAP was prepared at a density of 2 × 10^6 cells/mL. 100 μL of the cell suspension was evenly dropped onto the surface of the thyroid acellular scaffold, which was then placed in a 24-well culture plate to construct the 3D *in vitro* tumor model. After seeding, the scaffolds were left undisturbed in the incubator for 2 h to allow for sufficient cell adhesion. After 2 h, 5 mL of fresh medium was added to each well to ensure the scaffold was fully submerged, and long-term culture was conducted at 37 °C with 5% CO2. The medium was changed every 2 days.

For the conventional 2D culture control group, the same volume (100 μL) of cell suspension was directly added to a 24-well plate to ensure the initial cell number in each well was consistent with the 3D group, and 5 mL of medium was added for culture. Samples were collected from both the 3D culture group and the 2D control group on days 5, 10, and 15 for subsequent analysis.

### 2.7 Cell proliferation and viability assays

#### 2.7.1 Cell proliferation assessment

To quantify the number of thyroid cancer cells in the acellular scaffold, a standard curve was first established. BCPAP cell suspensions with known cell numbers (1 × 10^5, 2 × 10^5, 5 × 10^5, 1 × 10^6, 2 × 10^6, 4 × 10^6, 8 × 10^6, and 1.6 × 10^7 cells) were prepared. DNA was extracted from each group using a DNA extraction kit, and the DNA content was measured. A standard curve was plotted with cell number as the X-axis and DNA content as the Y-axis to obtain a linear equation representing the relationship between cell number and DNA content. On days 5, 10, and 15 of culture, samples were taken from the 3D scaffold group. DNA was extracted from the cells within the scaffold, and the DNA content was measured. The measured DNA content was substituted into the linear equation of the standard curve to calculate the number of cells in the scaffold, thereby assessing cell proliferation within the 3D scaffold.

#### 2.7.2 Cell viability assay

On days 5, 10, and 15, cell viability was measured using the CCK-8 kit (Dojindo). 100 μL of culture medium was taken from both the experimental and control groups, and 10 μL of CCK-8 reagent was added. After mixing, the samples were incubated for 2 h. After incubation, 100 μL of the reaction solution was transferred to a 96-well plate, and the absorbance (OD value) was measured at 450 nm using a microplate reader. The experiment was repeated 3 times to ensure reliability.

#### 2.7.3 Live/dead staining

To visually compare cell viability, both the 3D cell-laden scaffolds and the 2D control cultures were assessed on day 15 using a Live/Dead Viability/Cytotoxicity Kit (Thermo Fisher Scientific). Samples were washed with PBS and incubated in a solution containing 2 μM Calcein-AM and 4 μM Propidium Iodide (PI) in serum-free DMEM for 30 min at 37 °C in the dark. After incubation, the samples were gently washed again with PBS and immediately observed under a laser scanning confocal microscope (Leica, STELLARIS 5). Live cells with intact plasma membranes convert the non-fluorescent Calcein-AM into green fluorescent calcein. Dead cells with compromised membranes are permeable to PI, which binds to nucleic acids and emits red fluorescence. For quantitative analysis, three random fields of view were captured for each sample (n = 3). The number of live (green) and dead (red) cells was counted using ImageJ software (NIH, United States). Cell viability was calculated as: Viability (%) = [Number of live cells/(Number of live cells + Number of dead cells)] × 100%.

#### 2.7.4 Apoptosis assay

To compare apoptosis rates, BCPAP cells were cultured in the 3D TAS model and standard 2D plates for 15 days. Cells were then harvested (trypsinization for 2D, and gentle agitation followed by collagenase digestion for 3D scaffolds) and washed with cold PBS. Apoptosis was assessed using the Annexin V-FITC/PI Apoptosis Detection Kit (BD Biosciences, United States) according to the manufacturer’s protocol. Briefly, cells were resuspended in 1X binding buffer, stained with Annexin V-FITC and Propidium Iodide (PI), and incubated in the dark for 15 min. Stained cells were immediately analyzed by flow cytometry (BD FACSCanto II, BD Biosciences). The percentages of viable (Annexin V-/PI-), early apoptotic (Annexin V+/PI-), late apoptotic (Annexin V+/PI+), and necrotic (Annexin V-/PI+) cells were quantified using FlowJo software (Tree Star, United States).

### 2.8 Spatial distribution and infiltration analysis of cells in the 3D scaffold

To evaluate the infiltration of thyroid cancer cells into the acellular scaffold, immunofluorescence staining was performed on days 5, 10, and 15. First, cell-laden scaffolds were fixed with 4% paraformaldehyde and permeabilized with a solution containing Triton X-100. To clearly visualize the scaffold’s microstructure, it was specifically labeled with an anti-Collagen I primary antibody, followed by incubation with a green fluorescent secondary antibody (Alexa Fluor 488). Finally, cell nuclei were counterstained with DAPI (5 μg/L) to show the spatial location of cells within the scaffold. The stained scaffolds were observed under a laser scanning confocal microscope (Leica, STELLARIS 5). Z-stack scanning was performed with a 10 μm interval to record cell distribution at different depths (10 μm, 50 μm, 100 μm), generating a cell distribution map within the scaffold. ImageJ software (NIH, United States) was used to analyze the images at each depth level and calculate the number of cells at depths of 10 μm, 50 μm, and 100 μm. To ensure counting accuracy, 5 random fields of view were selected at each depth level for cell counting, and the average cell number was calculated. The relative infiltration rates at 50 μm and 100 μm were then calculated by comparing them to the cell number at the surface layer (10 μm depth), using the formulas: Relative Infiltration Rate (50 μm) = (N_50μm_/N_10μm_) × 100%, and Relative Infiltration Rate (100 μm) = (N_100μm_/N_10μm_) × 100%. Finally, the relative infiltration rates at different time points and depths were compared and analyzed.

### 2.9 Gene expression analysis of thyroid cancer-related genes

To detect the expression levels of the BRAF V600E gene and the epithelial-mesenchymal transition (EMT)-related genes E-cadherin (CDH1), N-cadherin (CDH2), and Vimentin (VIM), thyroid cancer cells cultured on the decellularized thyroid scaffold were collected on days 5, 10, and 15. Total RNA was extracted using TRIzol reagent (Invitrogen), and its concentration and purity were measured with a Nanodrop 2000 (Thermo Fisher Scientific). After removing genomic DNA contamination with DNase I (Thermo Fisher Scientific), 1 μg of total RNA was reverse-transcribed into cDNA using a reverse transcription kit (Thermo Fisher Scientific). Real-time quantitative PCR (qPCR) was performed using SYBR Green Master Mix (Applied Biosystems) on a StepOnePlus™ Real-Time PCR System (Applied Biosystems). The qPCR program was: 95 °C for 10 min, followed by 40 cycles of 95 °C for 15 s, 60 °C for 30 s, and 72 °C for 30 s. Relative gene expression was calculated using the 2^(−ΔΔCt) method, with GAPDH as the internal reference gene for normalization. The primer sequences for each gene are listed in Table 1. All experiments were repeated three times.

**TABLE 1:** Primer sequences.

Gene	Forward primer (5′-3′)	Reverse primer (5′-3′)
GAPDH	GCA​CCG​TCA​AGG​CTG​AGA​AC	TGG​TGA​AGA​CGC​CAG​TGG​A
BRAF V600E	TGA​TTT​TGG​TCT​AGC​TAC​AGA​G	CCT​CAA​TTC​TTA​CCA​TCC​ACA
E-cadherin	TGC​TCT​TCT​TCT​GCT​CAT​GTT​CCT​G	TCT​TCT​CCA​CCG​CCT​TCC​TCA​TC
N-cadherin	AGG​AGT​CAG​TGA​AGG​AGT​CAG​CAG	TTC​TGG​CAA​GTT​GAT​TGG​AGG​GAT​G
Vimentin	CCT​TCG​TGA​ATA​CCA​AGA​CCT​GCT​C	AAT​CCT​GCT​CTC​CTC​GCC​TTC​C

### 2.10 Immunofluorescence staining for EMT markers and HIF-1α

After 15 days of culture, 2D cultured cells (on glass coverslips) and 3D cell-laden scaffolds were fixed with 4% paraformaldehyde for 30 min 3D scaffolds were then cryopreserved in sucrose gradients before being embedded in OCT compound and sectioned into 10 μm thick slices. 2D coverslips and 3D sections were permeabilized with 0.2% Triton X-100 for 15 min and blocked with 5% goat serum for 1 h. Samples were then incubated overnight at 4 °C with primary antibodies: anti-E-cadherin (1:200, Cell Signaling Technology, #3195), anti-N-cadherin (1:200, Cell Signaling Technology, #13116), anti-Vimentin (1:200, Cell Signaling Technology, #5741), or anti-HIF-1α (1:200, Abcam, ab179483). After washing, samples were incubated with appropriate Alexa Fluor-conjugated secondary antibodies (1:500, Thermo Fisher Scientific) for 1 h at room temperature. Nuclei were counterstained with DAPI (5 μg/L). Images were captured using a laser scanning confocal microscope (Leica, STELLARIS 5). For quantitative analysis, the Mean Fluorescence Intensity (MFI, in arbitrary units, A.U.) of E-cadherin, N-cadherin, and Vimentin, and the nuclear fluorescence intensity of HIF-1α were measured from at least five independent fields of view per condition using ImageJ software (NIH, United States). Background-subtracted intensities were calculated and compared between 2D and 3D groups.

### 2.11 Drug sensitivity evaluation of the 3D tumor model

#### 2.11.1 Drug treatment

On day 10 of culture, the drug resistance of cells in 2D and 3D cultures was tested against cisplatin (Sigma) and Vemurafenib (Selleck Chemicals). For cisplatin treatment, different concentrations of cisplatin solution were added to the culture medium to achieve final concentrations of 5, 10, 20, 40, and 80 μM. For Vemurafenib treatment, the final concentrations were 0.5, 1, 2, 5, and 10 μM. The drugs were dissolved in sterile PBS and diluted to the target concentrations. The control group received an equal volume of PBS. After treatment, the samples were incubated for an additional 48 h at 37 °C with 5% CO_2_.

#### 2.11.2 Cell viability assay and IC50 calculation

After 48 h of drug treatment, the viability of cells in each group was measured using the CCK-8 reagent (Dojindo, Japan). 10% volume of CCK-8 reagent was added to each culture well, and the plates were incubated for 2 h at 37 °C with 5% CO2. The absorbance (OD450 value) of each sample was then measured at a wavelength of 450 nm using a microplate reader. Each sample was tested in triplicate. The OD450 values of all concentration groups were normalized to the percentage of viability relative to the control group (no drug treatment), calculated as: Viability (%) = (OD450 of experimental group/OD450 of control group) × 100%. A dose-response curve was plotted with drug concentration (x-axis) versus viability (y-axis). A non-linear fit was performed using the Boltzmann equation: y = (A1 - A2)/(1 + e^((x - x_0_)/dx)) + A2, where y is viability and x is the log of the drug concentration. The IC50 value (the drug concentration corresponding to 50% viability) was obtained from the fitted curve to evaluate and compare the drug resistance of cells under different culture conditions.

#### 2.11.3 Combination therapy for overcoming drug resistance

To test the hypothesis that drug resistance in the 3D model was mediated by bypass signaling pathways, combination drug treatments were performed on day 10 of 3D culture. The following inhibitors were used: Vemurafenib (BRAF inhibitor, Selleck Chemicals), Trametinib (MEK inhibitor, Selleck Chemicals), and Defactinib (FAK inhibitor, Selleck Chemicals). Based on the IC50 value determined in Section 2.11.2, a concentration of 5 µM Vemurafenib was used. Four treatment groups were established: 1. Vehicle control (DMSO); 2. Vemurafenib (5 µM) alone; 3. Vemurafenib (5 µM) + Trametinib (2 µM); 4. Vemurafenib (5 µM) + Defactinib (2 µM). Cells were treated for 48 h. Cell viability was measured using the CCK-8 kit as determined in Section 2.11.2, and viability was expressed as a percentage relative to the vehicle control group.

## 3 Results

### 3.1 Successful preparation and characterization of the thyroid acellular scaffold (TAS)

#### 3.1.1 Histological and morphological analysis

**FIGURE 2:**
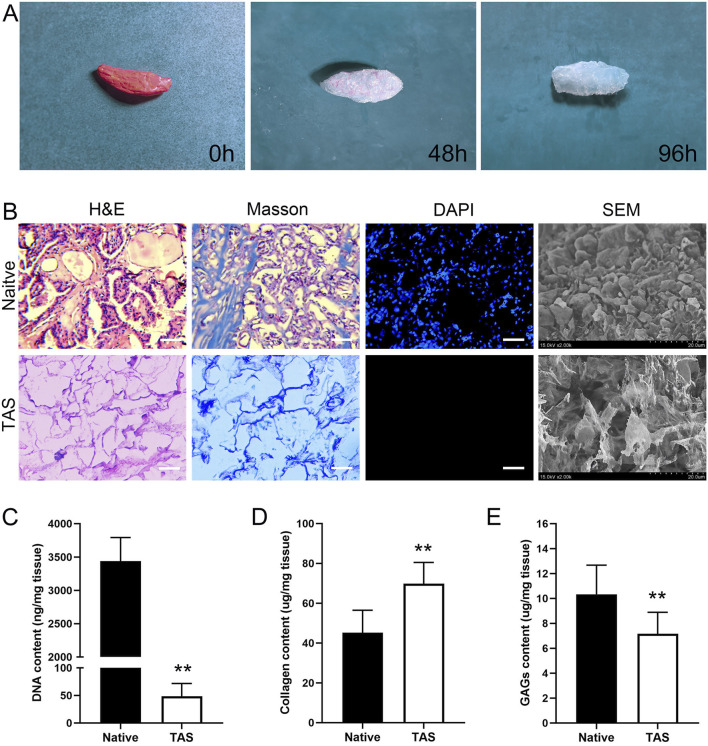
Characterization of the Thyroid Acellular Scaffold (TAS). **(A)** Macroscopic appearance of the rat thyroid tissue throughout the decellularization process, showing the transition from native tissue (0 h) to a translucent scaffold (96 h). **(B)** Histological and morphological comparison between native thyroid tissue and the TAS. H&E and Masson’s trichrome staining demonstrate the preservation of the extracellular matrix structure and collagen fibers (blue) after cell removal. DAPI staining confirms the complete removal of cell nuclei in the TAS. Scanning electron microscopy (SEM) images reveal the porous, interconnected fibrous network of the TAS compared to the cellular surface of the native tissue. Scale bars: 100 µm. **(C–E)** Quantitative biochemical analysis of the native tissue and TAS. Bar graphs show a significant reduction in **(C)** DNA content, a relative increase in **(D)** collagen content, and the retention of **(E)** glycosaminoglycans (GAGs) after decellularization. Data are presented as mean ± standard deviation. **p < 0.01 compared to native tissue.

As shown in Figure 2A, after decellularization, the originally pinkish thyroid tissue turned into a translucent white scaffold, macroscopically indicating that cellular components had been removed. H&E staining results showed that, compared to the native thyroid tissue which contained a large number of cell nuclei, no nuclear structures were observed in the decellularized scaffold, with only the outline of the extracellular matrix (ECM) remaining (Figure 2B). Masson’s trichrome staining further confirmed that the blue-stained areas representing collagen were well-preserved and evenly distributed in the scaffold, indicating that the main collagen network structure was not damaged during the decellularization process (Figure 2B). DAPI staining provided the most direct evidence for the removal of nuclei. While numerous bright blue fluorescent nuclei were visible in the native tissue, there was a complete absence of fluorescent signals in the decellularized scaffold, confirming the thorough removal of nuclei (Figure 2B). Scanning electron microscopy (SEM) images visually demonstrated the 3D porous fibrous network structure of the scaffold, which mimics the native tissue microenvironment and is conducive to subsequent cell adhesion and growth (Figure 2B).

#### 3.1.2 Biochemical component quantitative analysis

Quantitative analysis results showed that, compared to native thyroid tissue (3,438 ± 354 ng/mg), the residual DNA content in the decellularized scaffold was significantly reduced to 49 ± 23 ng/mg (p < 0.01), which is below the biomaterial safety standard of 50 ng/mg, demonstrating the thoroughness of decellularization (Figure 2C). Due to the removal of cellular components, the relative content of collagen in the scaffold increased from 45.33 ± 11.60 μg/mg in native tissue to 70.17 ± 10.83 μg/mg (p < 0.01), indicating that the main structural protein was effectively preserved (Figure 2D). Meanwhile, the scaffold retained approximately 71% of sulfated glycosaminoglycans (sGAG), with its content decreasing from 10.33 ± 2.34 μg/mg in native tissue to 7.33 ± 1.51 μg/mg after decellularization (Figure 2E).

### 3.2 Analysis of key ECM proteins and proteomics

#### 3.2.1 Preservation of major ECM proteins

**FIGURE 3:**
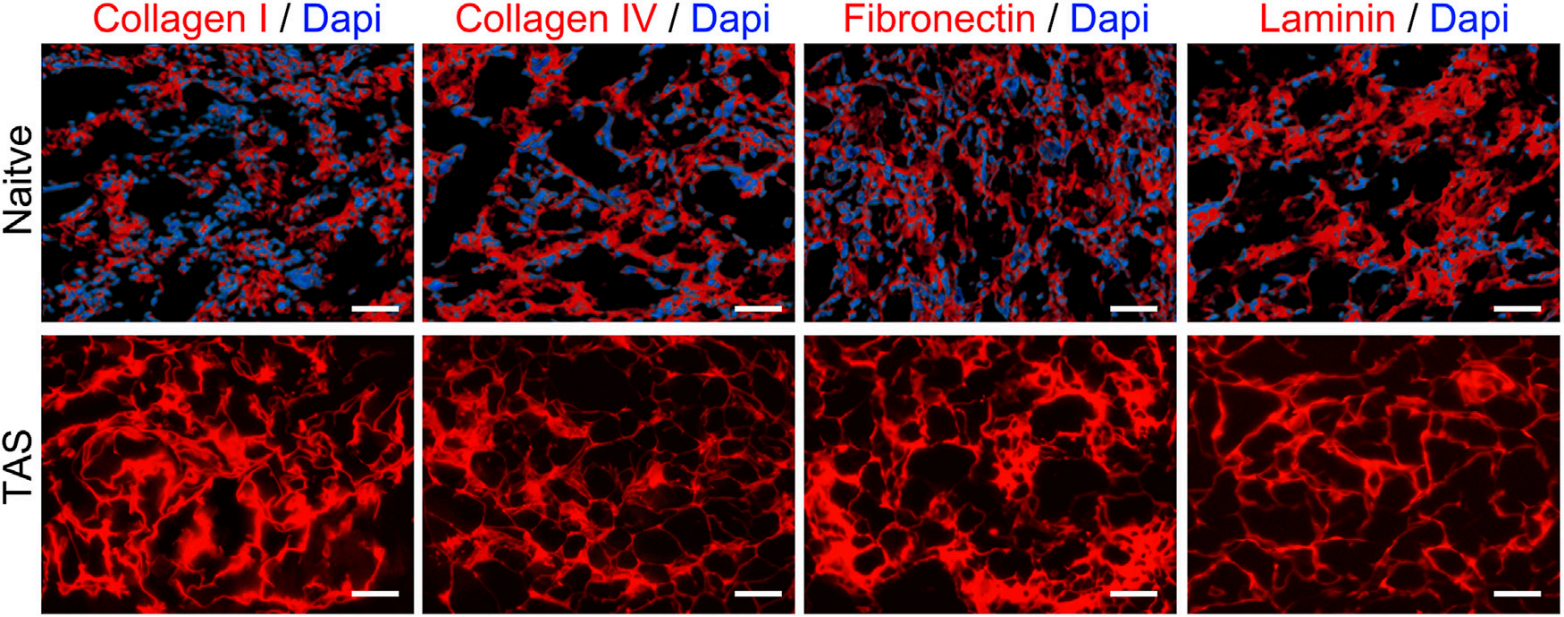
Preservation of key extracellular matrix (ECM) proteins in the Thyroid Acellular Scaffold (TAS). Immunofluorescence staining for major ECM components in native thyroid tissue (top row) and the TAS (bottom row). Staining shows the retention of Collagen I, Collagen IV, Fibronectin, and Laminin (all shown in red) after the decellularization process. In the native tissue, cell nuclei are counterstained with DAPI (blue). The absence of DAPI staining in the TAS confirms the successful removal of cells, while the preserved network structure of the ECM proteins indicates that the scaffold maintains its essential biochemical composition. Scale bars: 100 µm.

Immunofluorescence staining results showed that key ECM proteins widely expressed in native thyroid tissue, such as Collagen I, Collagen IV, Fibronectin, and Laminin, were well-preserved in the decellularized scaffold (Figure 3). These proteins exhibited a network-like distribution pattern in the scaffold, similar to that in native tissue, providing a structural and functional basis for constructing a biomimetic microenvironment.

#### 3.2.2 Proteomic analysis

**FIGURE 4:**
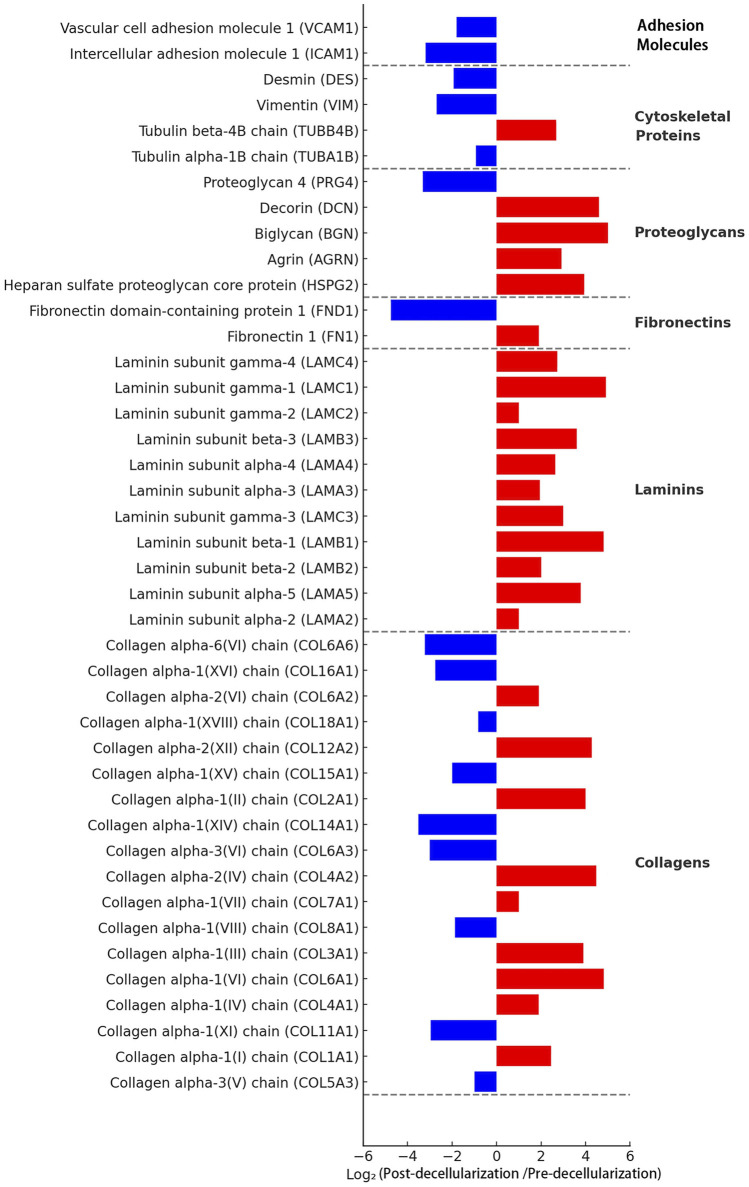
Proteomic analysis of the Thyroid Acellular Scaffold (TAS). The bar chart displays the log2 fold change of protein abundance in the TAS compared to native thyroid tissue, as determined by mass spectrometry. Proteins are categorized by function. Red bars indicate proteins that were retained or relatively enriched after decellularization (positive log2 fold change), while blue bars represent proteins that were significantly depleted or removed (negative log2 fold change). The analysis confirms the effective removal of cellular components, such as adhesion molecules (e.g., VCAM1, ICAM1) and cytoskeletal proteins (e.g., Vimentin), while demonstrating the excellent preservation of major extracellular matrix (ECM) structural proteins, including multiple subtypes of collagens, laminins, proteoglycans, and fibronectins. This proteomic profile underscores the successful creation of an ECM-rich scaffold suitable for 3D cell culture.

To comprehensively understand the changes in protein components during the decellularization process, we performed LC-MS/MS analysis. The results showed that the protein profile of the decellularized scaffold was significantly altered compared to native thyroid tissue (Figure 4). The analysis indicated that major extracellular matrix structural proteins were effectively preserved. For example, various types of collagen (e.g., COL1A1, COL3A1, COL4A1), laminin subunits (e.g., LAMA4, LAMB1, LAMC1), and fibronectin (FN1) had positive log2 ratios, indicating their abundance was stable or relatively increased after decellularization. Additionally, various proteoglycans (e.g., HSPG2, BGN, DCN) were also well-preserved.

Conversely, the vast majority of intracellular proteins were successfully removed. Intracellular cytoskeletal proteins (e.g., vimentin VIM, tubulin TUBA1B) and cell adhesion molecules (e.g., VCAM1, ICAM1) had large negative log2 ratios, indicating a significant decrease in their abundance after decellularization. These results confirm at the molecular level that our decellularization method effectively removes cellular components while maximally preserving the extracellular matrix components crucial for constructing tissue engineering models.

### 3.3 Growth factor retention in the decellularized scaffold

**FIGURE 5:**
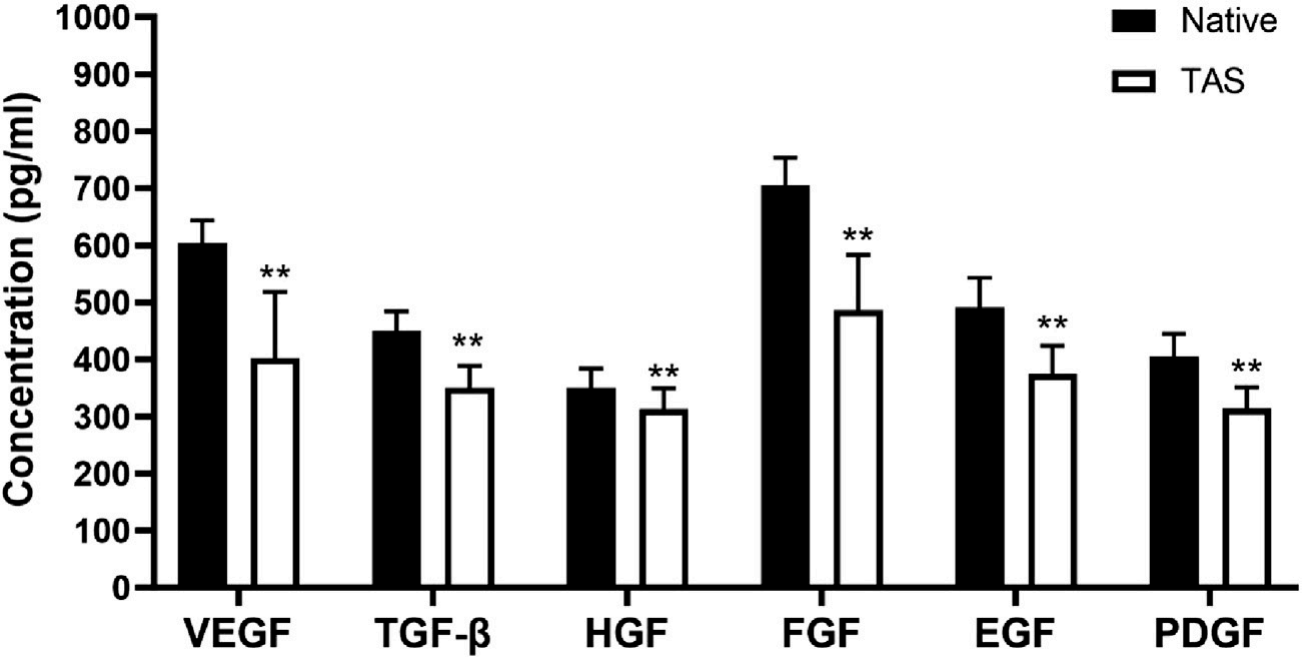
Retention of endogenous growth factors in the Thyroid Acellular Scaffold (TAS). The bar chart shows the concentrations (pg/mL) of key growth factors—Vascular Endothelial Growth Factor (VEGF), Transforming Growth Factor-β (TGF-β), Hepatocyte Growth Factor (HGF), Fibroblast Growth Factor (FGF), Epidermal Growth Factor (EGF), and Platelet-Derived Growth Factor (PDGF)—in native thyroid tissue and the TAS, as quantified by ELISA. Although the decellularization process resulted in a significant reduction in the concentration of all measured growth factors, the TAS successfully retained a substantial amount of these bioactive molecules. Data are presented as mean ± standard deviation. **p < 0.01 compared to native tissue.

ELISA quantitative detection results showed that native thyroid tissue is rich in endogenous growth factors. After the decellularization process, although some growth factors were lost, a considerable amount of key growth factors was retained in the decellularized scaffold (Figure 5). Specifically, compared to native tissue, the decellularized scaffold retained approximately 67% of VEGF, 78% of TGF-β, 89% of HGF, 69% of FGF, 76% of EGF, and 78% of PDGF. These preserved growth factors can provide necessary biological signals to the seeded tumor cells, stimulating their proliferation and functional activities, thereby better mimicking the *in vivo* tumor growth microenvironment.

### 3.4 Mechanical properties of the thyroid acellular scaffold

**FIGURE 6:**
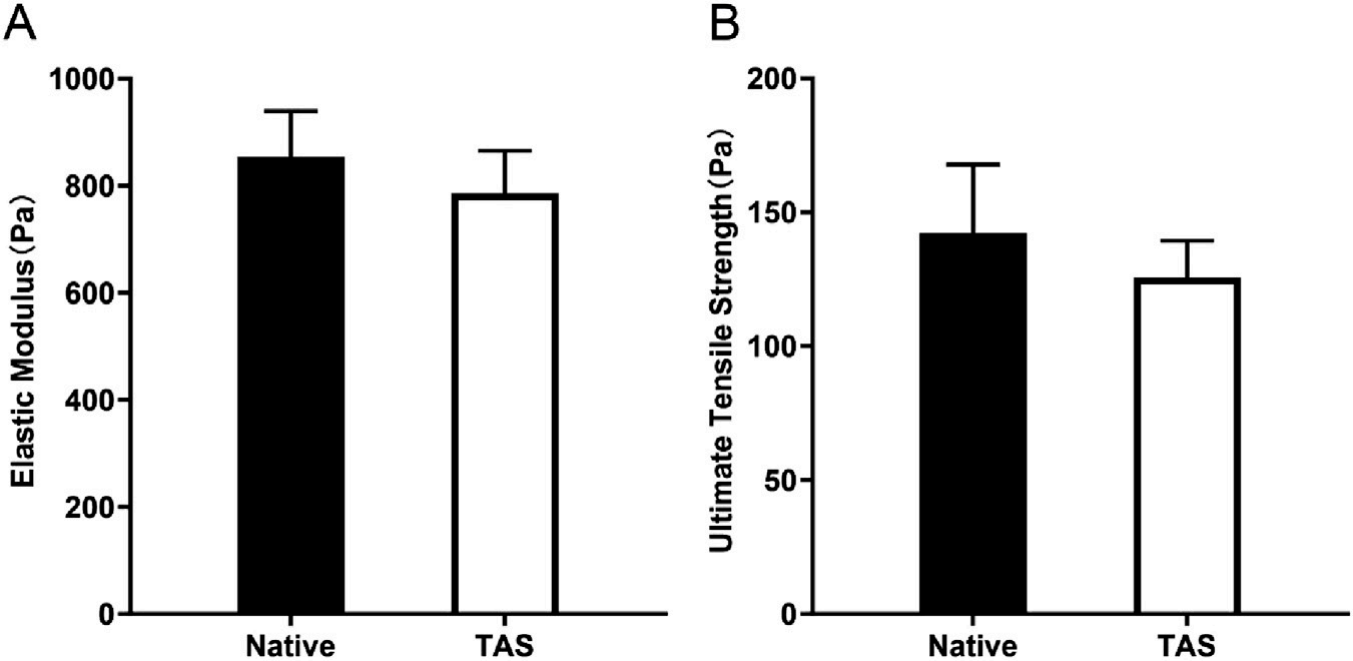
Mechanical properties of the Thyroid Acellular Scaffold (TAS). Bar charts comparing the **(A)** Elastic Modulus and **(B)** Ultimate Tensile Strength of native thyroid tissue and the TAS. The results indicate that while there was a slight decrease in both elastic modulus and ultimate tensile strength after decellularization, these differences were not statistically significant (p > 0.05). This demonstrates that the TAS retains mechanical properties comparable to the native tissue, ensuring its structural integrity for use as a 3D culture scaffold. Data are presented as mean ± standard deviation.

In mechanical property testing, the elastic modulus of the native thyroid tissue group ranged from 723 to 976 Pa, with an average of 853 Pa. The elastic modulus of the decellularized scaffold group ranged from 664 to 883 Pa, with an average of 786 Pa. Although the elastic modulus slightly decreased after decellularization, the difference between the two groups was not statistically significant (p > 0.05) (Figure 6A). Furthermore, the ultimate tensile strength of the native thyroid tissue group ranged from 113 to 183 Pa, with an average of 142 Pa, while that of the decellularized scaffold group ranged from 110 to 145 Pa, with an average of 126 Pa. Similarly, the difference in ultimate tensile strength between the two groups was not statistically significant (p > 0.05) (Figure 6B). Comprehensive analysis indicates that while the decellularization process had some effect on the mechanical properties (including elastic modulus and ultimate tensile strength) of the thyroid scaffold, its mechanical performance remained at a high level, sufficient to meet the requirements for cell culture and tissue engineering applications.

### 3.5 Construction and culture of the 3D in vitro tumor model

In this study, we systematically evaluated the proliferation of BCPAP thyroid cancer cells in the 3D thyroid acellular scaffold versus a 2D culture environment using both CCK-8 and DNA quantification methods. The results showed that the 3D scaffold has a significant advantage in promoting cell proliferation, although the proliferation rate tends to stabilize during long-term culture.

**FIGURE 7:**
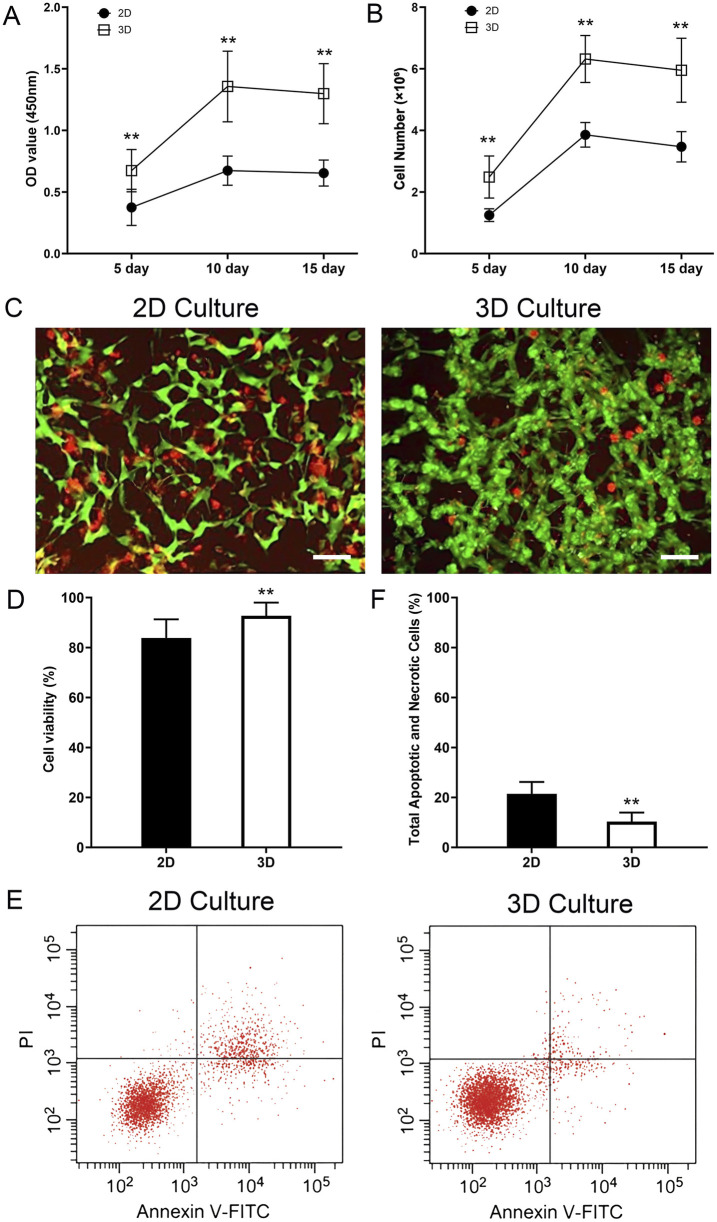
Enhanced proliferation, viability, and reduced apoptosis of thyroid cancer cells in the 3D tumor model. **(A)** Cell viability measured by CCK-8 assay at 5, 10, and 15 days. The optical density (OD) at 450 nm is significantly higher in the 3D culture group compared to the 2D group at all time points. **(B)** Cell number quantified by DNA content at 5, 10, and 15 days. The 3D scaffold supports a significantly higher number of cells compared to the 2D culture. Both groups show a plateau in proliferation after day 10. **(C)** Representative Live/Dead staining images of cells in 2D and 3D cultures on day 15. Live cells are stained green (Calcein-AM) and dead cells are stained red (Propidium Iodide). **(D)** Quantification of cell viability from Live/Dead staining, showing significantly higher viability in the 3D model. **(E)** Representative flow cytometry plots showing Annexin V-FITC and Propidium Iodide (PI) staining for cells cultured in 2D and 3D environments for 15 days. In each panel the lower left quadrant shows live cells, which are negative for both PI and annexin V-FITC, the upper left quadrant shows only PI positive cells, which are necrotic. The lower right quadrant shows annexin positive cells (early apoptotic) and the upper right quadrants shows annexin and PI positive cells (late apoptosis cells). **(F)** Quantification of the total percentage of apoptotic and necrotic cells (early apoptosis + late apoptosis + necrosis). The 3D model shows a significantly lower total cell death rate compared to the 2D control. Data are presented as mean ± standard deviation. Scale bars: 50 µm. **p < 0.01 compared to the 2D group.

The CCK-8 assay revealed that the cell viability in the 3D scaffold group was significantly higher than in the 2D group throughout the culture period (P < 0.05). In the first 10 days of culture, the cell proliferation activity in the 3D scaffold group increased rapidly, with the OD value rising from 0.67 ± 0.17 to 1.36 ± 0.29, which was significantly higher than the increase in the 2D group during the same period (from 0.38 ± 0.15 to 0.67 ± 0.12). However, between day 10 and day 15, the cell viability in the 3D group remained stable, with no significant change in the OD value (P > 0.05), indicating that the cells had entered a proliferation plateau phase. In contrast, the cell viability in the 2D group remained at a low level throughout the culture period and also showed no significant change between day 10 and day 15 (Figure 7A).

DNA quantification analysis further validated these findings. The cell number in the 3D scaffold group increased rapidly in the first 10 days, from (2.49 ± 0.68) × 10^6^ to (6.32 ± 0.76) × 10^6^, significantly higher than the increase in the 2D group during the same period, from (1.25 ± 0.21) × 10^6^ to (3.86 ± 0.40) × 10^6^ (P < 0.01). However, between day 10 and day 15, the cell number in the 3D group did not change significantly (P > 0.05), indicating that cell proliferation had reached a plateau. In contrast, the increase in cell number in the 2D group was smaller throughout the culture period and also tended to stabilize between day 10 and day 15 (Figure 7B).

To directly assess cell viability and further investigate the cellular state during the proliferation plateau phase, a Live/Dead staining assay was performed on both 2D and 3D cultures on day 15. The results revealed a significant advantage of the 3D microenvironment in maintaining long-term cell survival. As shown in Figure 7C, while both models contained viable cells, the 2D culture exhibited a noticeably higher proportion of dead cells (red fluorescence) compared to the 3D model. Critically, cells within the core of the 3D scaffold displayed robust viability, with a dense population of live cells (green fluorescence) and only sporadic dead cells. Quantitative analysis (Figure 7D) confirmed this observation, demonstrating that the cell viability in the 3D group (92.7% ± 5.3%) was significantly higher than in the 2D group (83.9% ± 7.5%) (p < 0.05). This provides strong evidence that the TAS provides a superior, pro-survival microenvironment that actively enhances the long-term health and viability of cancer cells.

To further investigate the mechanism behind this enhanced viability, we directly quantified apoptosis and necrosis using Annexin V-FITC/PI flow cytometry. The results clearly demonstrated that cells cultured in the 3D TAS model exhibit a significantly lower rate of spontaneous cell death compared to those in the 2D model (Figure 7E). Quantitative analysis (Figure 7F) showed that the total percentage of apoptotic and necrotic cells (including early apoptosis, late apoptosis, and necrosis) was significantly lower in the 3D group (10.4% ± 3.6%) compared to the 2D group (21.4% ± 4.8%) (p < 0.01). This new finding strongly supports our central hypothesis that the TAS provides a more biomimetic, pro-survival microenvironment, shielding cancer cells from the stress typically associated with artificial 2D culture conditions.

### 3.6 Spatial distribution and infiltration analysis of cells in the 3D scaffold

**FIGURE 8:**
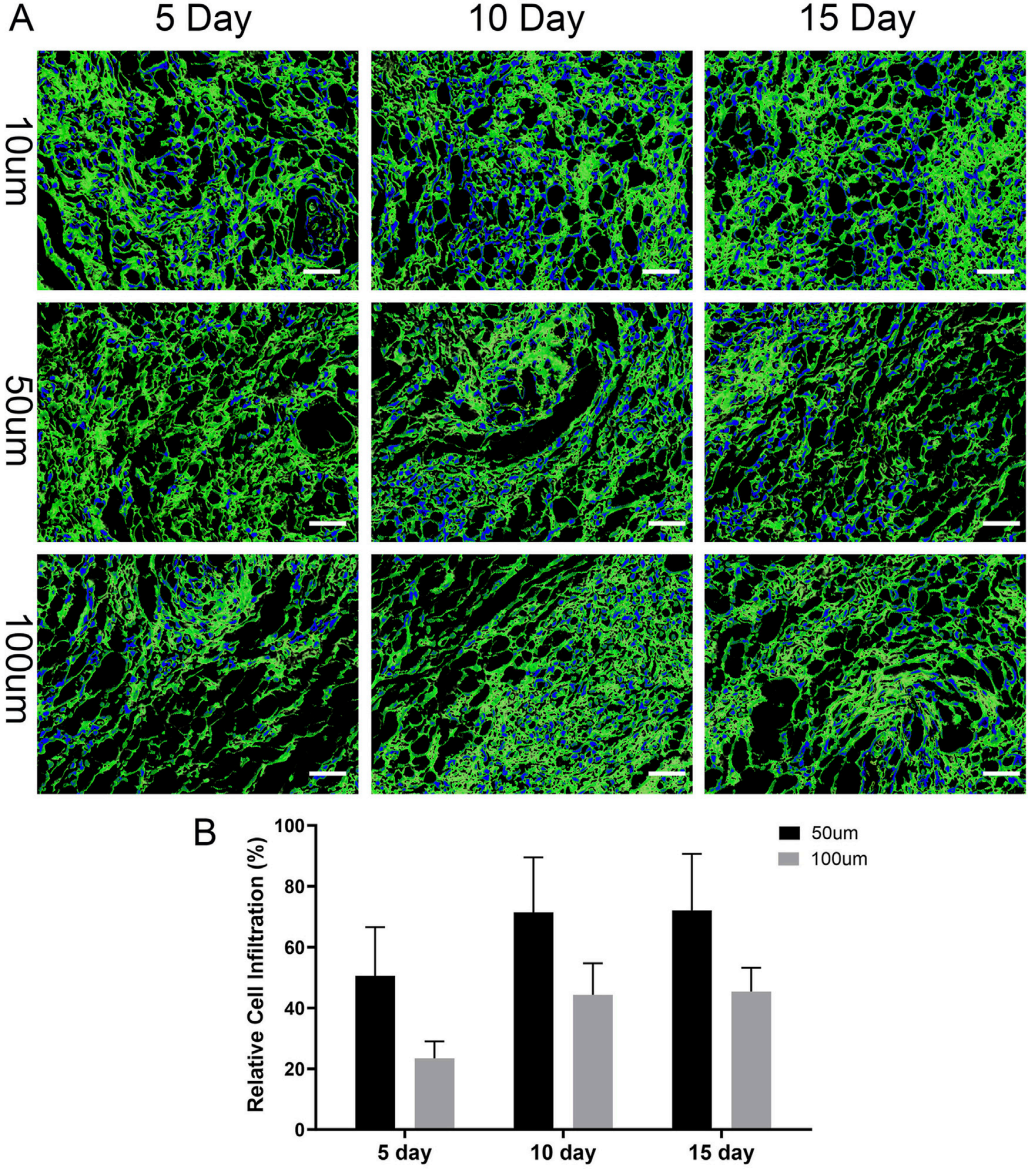
Time-dependent infiltration of thyroid cancer cells into the 3D scaffold. **(A)** Representative confocal microscopy images showing the spatial distribution of thyroid cancer cells within the scaffold at depths of 10, 50, and 100 µm after 5, 10, and 15 days of culture. The scaffold’s extracellular matrix was visualized by immunofluorescence staining for Collagen I (green), and cell nuclei were counterstained blue with DAPI. The images illustrate that cells progressively penetrate deeper into the scaffold over time. Scale bars: 100 µm. **(B)** Quantification of relative cell infiltration at 50 μm and 100 µm depths. The percentage is calculated relative to the cell population at the 10 µm surface layer. The data show a significant increase in cell penetration into the scaffold’s interior over the 15-day culture period, indicating the model supports cancer cell invasion. Data are presented as mean ± standard deviation.

On days 5, 10, and 15, the infiltration of BPCAP thyroid cancer cells into the thyroid acellular scaffold was analyzed using a laser scanning confocal microscope, and the relative infiltration rates at different depths were calculated. On day 5, BPCAP cells were mainly concentrated on the surface layer of the scaffold (10 μm depth), with relatively less infiltration into deeper layers (50 μm and 100 μm). Specifically, the relative infiltration rate at 50 μm depth was 50.6%, while at 100 μm depth it was 23.4%. This indicates that on day 5, most cells remained on the surface and in the shallow layers of the scaffold, with limited deep infiltration. On day 10, the infiltration ability of BPCAP cells into the scaffold was significantly enhanced. Although the cell density at the 10 μm depth remained high, the relative infiltration rates at 50 μm and 100 μm depths increased to 71.5% and 44.3%, respectively. Compared to day 5, the relative infiltration rates at 50 μm and 100 μm depths on day 10 were significantly increased (p < 0.01), indicating that cells gradually infiltrated deeper into the scaffold over time. By day 15, the cell infiltration situation showed no significant change compared to day 10. The relative infiltration rate at 50 μm depth was approximately 72.1%, and at 100 μm depth, it was about 45.4%. This suggests that from day 10 to day 15, the distribution of cells in the deeper layers of the scaffold tended to stabilize, and the infiltration ability reached a plateau phase (Figures 8A,B).

Statistical analysis shows that the infiltration ability of BPCAP thyroid cancer cells in the thyroid acellular scaffold increased with culture time, reaching a peak on day 10 and then stabilizing. Infiltration into the deeper layers of the scaffold (50 μm and 100 μm) was particularly significant. This result indicates that by days 10–15, the migration and diffusion abilities of the cells within the scaffold reached a relatively ideal and stable state, which is conducive to constructing a more uniform and mature *in vitro* thyroid cancer tumor model.

### 3.7 Gene expression analysis of thyroid cancer-related genes

#### 3.7.1 BRAF V600E gene expression level

**FIGURE 9:**
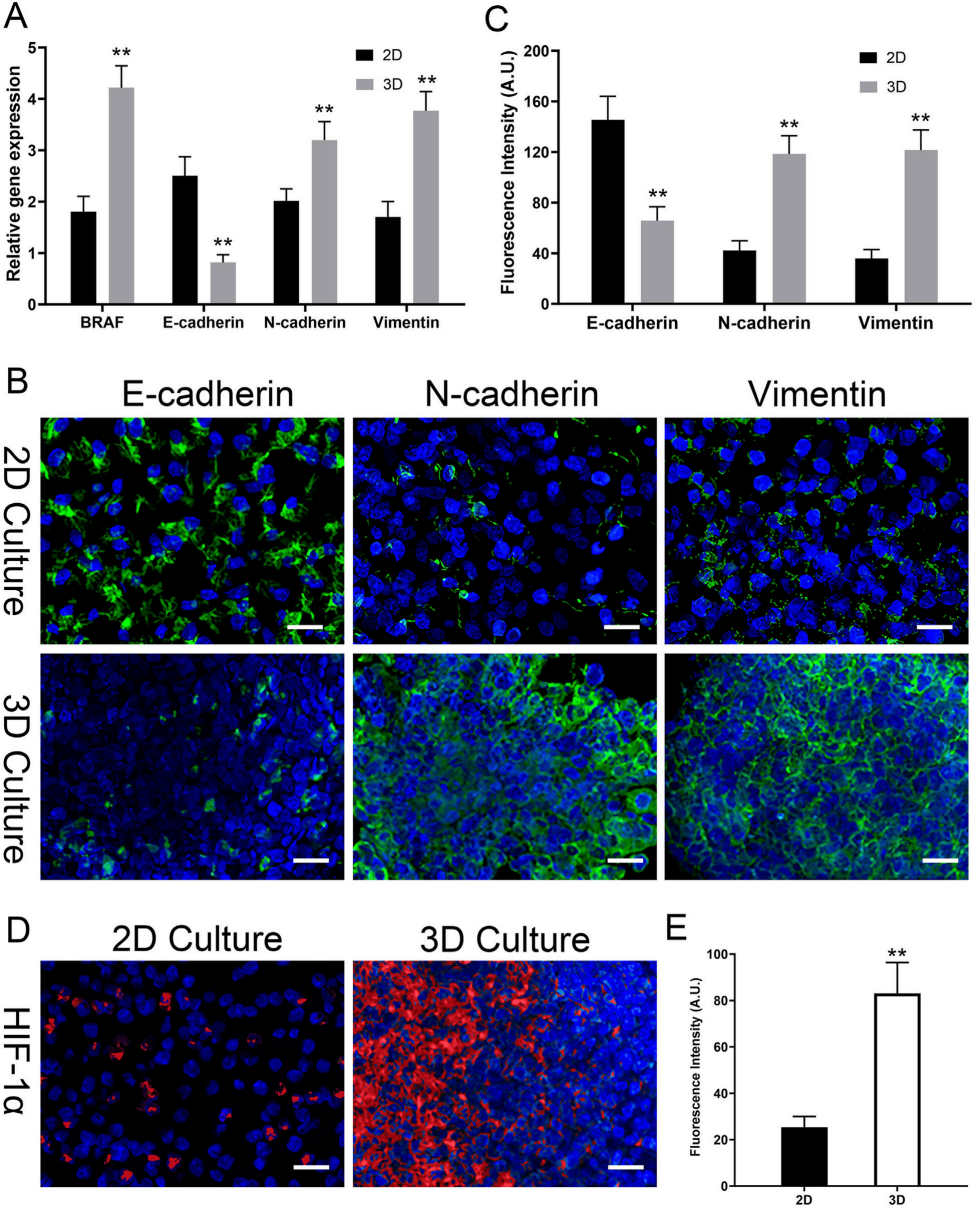
3D Model Induces EMT and Hypoxia at Gene and Protein Levels. **(A)** Relative gene expression of BRAF V600E and EMT markers (E-cadherin, N-cadherin, Vimentin) by qPCR. 3D culture upregulates BRAF V600E and induces an EMT-like gene signature. **(B)** Representative confocal immunofluorescence images for EMT markers (E-cadherin, N-cadherin, Vimentin; all in green) in 2D vs. 3D. 3D culture induces a protein-level switch from epithelial (E-cad high) to mesenchymal (N-cad/Vim high). **(C)** Quantitative analysis of the mean fluorescence intensity (MFI, in A.U.) for EMT markers from **(B)**. **(D)** Representative immunofluorescence images of HIF-1α (red) in 2D vs. 3D. Strong HIF-1α accumulation is seen only in the 3D model. All images are counterstained with DAPI (blue, nuclei). **(E)** Quantitative analysis of HIF-1α fluorescence intensity (MFI, in A.U.) from **(D)**. Data are presented as mean ± standard deviation. Scale bars: 50 µm. *p < 0.05, **p < 0.01 compared to the 2D group.

In the analysis of BRAF V600E gene expression, the expression of the BRAF V600E gene in thyroid cancer cells in the 3D scaffold group was significantly higher than in the 2D group. Specifically, the relative expression level of the BRAF V600E gene in the 3D scaffold group was 4.2 ± 0.4, whereas in the 2D group it was only 1.8 ± 0.3, resulting in a 2.3-fold expression ratio, a statistically significant difference (P < 0.01) (Figure 9A). This result indicates that thyroid cancer cells exhibit stronger BRAF V600E gene expression activity in the three-dimensional decellularized scaffold environment compared to the two-dimensional flat culture environment, suggesting that the 3D scaffold may more effectively mimic the gene regulatory characteristics of the tumor microenvironment.

#### 3.7.2 Epithelial-mesenchymal transition (EMT)-Related gene expression levels

In the analysis of EMT-related gene expression, there were significant differences between the 3D scaffold group and the 2D group. Specifically, in the 3D scaffold group, the expression of the epithelial marker E-cadherin (CDH1) gene was significantly reduced, with a relative expression level of 0.8 ± 0.2, whereas in the 2D group, the expression level of this gene was 2.5 ± 0.4, showing a significant inhibitory effect (P < 0.05). Conversely, the expression of the mesenchymal markers N-cadherin (CDH2) and Vimentin (VIM) genes was significantly upregulated in the 3D scaffold group, at 3.2 ± 0.4 and 3.8 ± 0.4, respectively, while the expression levels of these genes were relatively low in the 2D group, at 2.0 ± 0.2 and 1.7 ± 0.3, respectively (P < 0.05) (Figure 9A). These results indicate that the three-dimensional decellularized thyroid scaffold can significantly influence the phenotypic transition of thyroid cancer cells, inhibiting epithelial characteristics while promoting the enhancement of mesenchymal phenotypes. The activation of this EMT phenomenon was particularly significant in the 3D scaffold group, further supporting the potential advantages of the 3D scaffold in simulating the tumor microenvironment.

### 3.8 Protein-level validation of EMT and confirmation of a hypoxic microenvironment

To confirm that the gene-level changes in EMT markers translated to the protein level, we performed immunofluorescence staining on cells cultured for 15 days. As shown in Figure 9B, cells in 2D culture exhibited strong E-cadherin staining at cell junctions, with weak expression of N-cadherin and Vimentin. In stark contrast, cells within the 3D TAS model showed a clear phenotypic switch: E-cadherin expression was markedly reduced, while the expression of mesenchymal markers N-cadherin and Vimentin was significantly upregulated. Quantitative analysis of the mean fluorescence intensity (MFI) robustly confirmed these visual observations (Figure 9C). The MFI for E-cadherin in the 2D group (145.4 ± 18.7 A.U.) was significantly higher than in the 3D group (65.7 ± 11.0 A.U., p < 0.01). Conversely, the MFIs for N-cadherin (42.1 ± 7.7 A.U.) and Vimentin (35.7 ± 7.2 A.U.) in the 2D group were significantly lower than those in the 3D group (118.4 ± 14.4 A.U. and 121.4 ± 15.9 A.U., respectively, p < 0.01). This provides direct quantitative and visual evidence that the 3D microenvironment drives a complete EMT.

Furthermore, we investigated our hypothesis that the 3D model develops a hypoxic microenvironment. We stained for hypoxia-inducible factor 1-alpha (HIF-1α), a master regulator of the hypoxic response (Figure 9D). In the 2D control group, no significant HIF-1α nuclear signal was detected, indicating a fully oxygenated environment. However, within the 3D scaffold, a strong and distinct accumulation of HIF-1α in the cell nuclei was observed. Quantitative analysis of the nuclear signal intensity (Figure 9E) confirmed this finding, showing a significantly higher nuclear MFI in the 3D group (83.0 ± 13.3 A.U.) compared to the 2D controls (25.3 ± 4.7 A.U., p < 0.01). This provides direct evidence of a hypoxic microenvironment that is absent in the 2D model.

### 3.9 Drug sensitivity evaluation of the 3D tumor model

In this study, we seeded thyroid cancer cells onto a thyroid acellular scaffold to construct an *in vitro* thyroid cancer tumor model and evaluated its drug tolerance. The 3D scaffold group consisted of thyroid cancer cells seeded onto the scaffold, while the 2D group consisted of flat-cultured thyroid cancer cells. On day 10, we treated both the 3D and 2D groups with different concentrations of Cisplatin and Vemurafenib and measured cell viability using the CCK-8 method. By fitting the dose-response curves with the Boltzmann equation, we obtained the IC50 values for each group to assess drug tolerance.

#### 3.9.1 Cisplatin drug resistance results

In the 2D group, cell viability decreased significantly with increasing concentrations of cisplatin. Specifically, at a cisplatin concentration of 5 μM, cell viability was 82.7% ± 12.1%. As the drug concentration increased, viability gradually decreased to 46.1% ± 11.3% at 20 μM and further to 29.8% ± 3.2% at 40 μM. At the highest concentration of 80 μM, cell viability dropped to its lowest at 15.4% ± 4.8%. Non-linear fitting of the viability curve yielded an IC50 value of 17.2 ± 2.7 μM for the 2D group, indicating that cisplatin has a strong cytotoxic effect on flat-cultured thyroid cancer cells.

**FIGURE 10:**
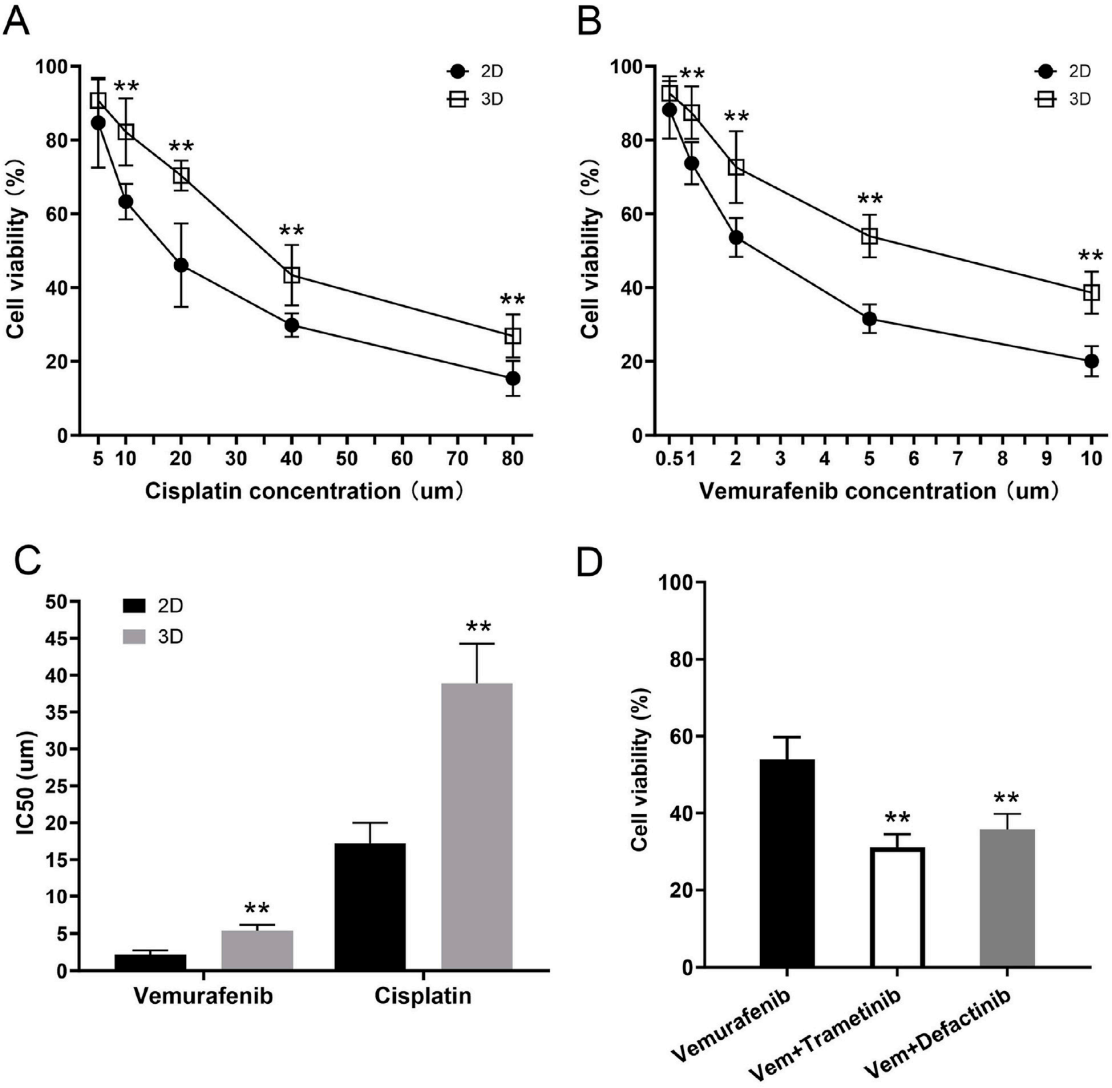
Increased drug resistance of the 3D tumor model and its reversal by combination therapy. **(A,B)** Dose-response curves showing the cell viability of thyroid cancer cells in 2D and 3D cultures after 48 h of treatment with varying concentrations of **(A)** Cisplatin and **(B)** Vemurafenib. At all tested concentrations, cells cultured in the 3D model demonstrated significantly higher viability compared to those in the 2D monolayer culture. **(C)** Comparison of the half-maximal inhibitory concentration (IC50) values for Vemurafenib and Cisplatin. The 3D tumor model exhibited significantly higher IC50 values for both drugs, confirming its enhanced resistance to both conventional chemotherapy and targeted therapy. **(D)** Combination therapy to overcome Vemurafenib resistance in the 3D model. Cells were treated for 48 h with Vehicle (Control), Vemurafenib (Vem, 5 μM) alone, or in combination with either Trametinib (MEKi, 2 µM) or Defactinib (FAKi, 2 µM). The combination therapies significantly reduced cell viability compared to Vemurafenib alone. Data are presented as mean ± standard deviation. **p < 0.01 (compared to the 2D group in **(A–C)**; compared to the Vemurafenib-only group in **(D** **)**).

Compared to the 2D group, the 3D scaffold group (thyroid cancer cells seeded on the scaffold) exhibited higher resistance to cisplatin. At a low concentration (5 μM) of cisplatin, the viability of the 3D scaffold group was 90.7% ± 5.7%, significantly higher than the 2D group. When the cisplatin concentration was increased to 20 μM, cell viability decreased to 70.4% ± 4.1%, but was still higher than the 2D group at the same concentration. As the cisplatin concentration further increased, viability was 43.4% ± 8.2% at 40 μM and dropped to 26.9% ± 5.8% at 80 μM. The fitted curve yielded an IC50 value of 38.9 ± 5.4 μM for the 3D scaffold group, which is higher than that of the 2D group, suggesting that the protective effect of the thyroid acellular scaffold may enhance the cells’ resistance to cisplatin (Figures 10A, C).

#### 3.9.2 Vemurafenib drug resistance results

In the 2D group, Vemurafenib also showed a significant inhibitory effect on flat-cultured thyroid cancer cells. As the Vemurafenib concentration increased from 0.5 μM to 10 μM, cell viability decreased from 88.2% ± 7.8% to 20.1% ± 4.1%. At 1 μM, viability was 73.7% ± 5.7%, and at 2 μM and 5 μM, viability was 53.6% ± 5.3% and 31.6% ± 3.9%, respectively. Curve fitting yielded an IC50 value of 2.2 ± 0.5 μM for the 2D group, indicating that Vemurafenib has a strong inhibitory effect on flat-cultured thyroid cancer cells at low concentrations.

The 3D scaffold group cells showed significant resistance to Vemurafenib treatment. At the lowest concentration (0.5 μM), the viability of the 3D scaffold group was 92.7% ± 4.6%, almost unaffected by the drug. When the concentration was increased to 1 μM, viability decreased to 87.4% ± 7.1%, but was still significantly higher than the 2D group. Similarly, as the Vemurafenib concentration increased, the viability of the 3D scaffold group gradually decreased to 72.6% ± 9.7%, 53.9% ± 5.8%, and 38.6% ± 5.7% at concentrations of 2, 5, and 10 μM, respectively. Non-linear fitting yielded an IC50 value of 5.4 ± 0.8 μM for the 3D scaffold group, significantly higher than the 2D group, indicating that the thyroid acellular scaffold has a protective effect on BRAFV600E-mutated thyroid cancer cells, thereby increasing their resistance to Vemurafenib (Figures 10B, C).

#### 3.9.3 Combination therapy with MEK or FAK inhibitors overcomes vemurafenib resistance in the 3D model

To investigate the mechanism of this acquired resistance, we explored combination therapies. We hypothesized that the 3D microenvironment activated bypass signaling pathways, such as the MEK/ERK pathway or the FAK-mediated integrin signaling pathway. We treated the 3D cultures with Vemurafenib (5 µM) alone or in combination with a MEK inhibitor (Trametinib, 2 µM) or a FAK inhibitor (Defactinib, 2 µM). As shown in the Figure 10D, treatment with Vemurafenib (5 µM) alone confirmed our previous findings, reducing cell viability to only 53.9% ± 5.8%. Critically, the combination of Vemurafenib with the MEK inhibitor (Trametinib, 2 µM) resulted in a dramatic and significant reduction in cell viability to 31.2% ± 3.3% (p < 0.01 vs. Vemurafenib alone). Similarly, the combination of Vemurafenib with the FAK inhibitor (Defactinib, 2 µM) also led to a significant enhanced cell kill, reducing viability to 35.8% ± 4.0% (p < 0.01 vs. Vemurafenib alone). These results strongly suggest that the CAM-DR observed in our 3D model is co-driven by the activation of both the MEK/ERK and FAK signaling pathways, and that co-targeting these pathways can effectively overcome the resistance.

## 4 Discussion

The global incidence of thyroid cancer continues to rise, making it the most common endocrine malignancy and posing a significant challenge to clinical diagnosis, treatment, and basic research (Pizzato et al., 2022). For a long time, our understanding of thyroid cancer biology and the development of anti-tumor drugs have largely relied on two-dimensional (2D) monolayer cell culture models. However, cells grown on these flat, rigid plastic surfaces lose the complex three-dimensional (3D) topology, close intercellular communication, and dynamic interactions with the extracellular matrix (ECM) that they experience in their native tissue (Langhans, 2018). This fundamental simplification in the model leads to inherent deficiencies in mimicking the true proliferation patterns, invasive potential, gene expression profiles, and drug responses of tumor cells (Jensen and Teng, 2020). This results in a significant gap between preclinical research and clinical trial outcomes, known as the “preclinical-to-clinical” translation failure (Millen et al., 2023). Therefore, developing 3D *in vitro* models that can highly mimic the *in vivo* tumor microenvironment (TME) has become a critical bottleneck to overcome in oncology research (Miserocchi et al., 2021; Brandenberg et al., 2020). In this study, we focused on the core component of the TME—the ECM. We innovatively used rat thyroid tissue and, through a refined decellularization technique, successfully prepared a thyroid acellular scaffold (TAS) that retains its native structure and bioactivity. Based on this, we constructed a 3D *in vitro* model of human thyroid cancer cells (BCPAP). This study systematically verified the significant superiority of this model over traditional 2D models in terms of morphology, biological function, and drug sensitivity, demonstrating its great potential as a high-fidelity research platform.

The first step of our study, and the foundation for building a highly biomimetic 3D model, was the preparation of a high-quality acellular scaffold. An ideal tissue engineering scaffold must not only provide structural support for cells but, more importantly, must offer a microenvironment rich in complex biochemical signals and physical cues to guide cell behavior (Chaudhuri et al., 2020). Compared to synthetic or semi-synthetic scaffolds made from one or a few purified ECM proteins, dECM scaffolds derived from the target organ itself have unparalleled advantages (Choi et al., 2023; A et al., 2020). They can maximally preserve the unique ECM components of a specific tissue, the nano-scale fibrous network topology, and the sequestered profile of growth factors and cytokines. This “tissue specificity” is crucial for simulating the microenvironment of a particular cancer (Crouigneau et al., 2024). This study employed a gentle chemical treatment method (a combination of SDS and Triton X-100) aimed at efficiently removing cellular components while maximally preserving the integrity of the ECM (Belviso et al., 2022). Our histological (H&E, Masson’s staining), immunofluorescence (DAPI), and biochemical quantitative analysis results collectively confirmed the success of this strategy. The residual DNA content was effectively controlled below the recognized safety standard for biomaterials (<50 ng/mg dry weight), virtually eliminating the risk of host immune rejection triggered by xenogeneic DNA and cellular antigens (Gilpin and Yang, 2017; Fazel et al., 2024). Furthermore, the absence of detectable detergent residues and the high cell viability (>95%) observed in TAS extract tests confirmed the excellent biocompatibility and biosafety of the prepared scaffold, further validating the robustness of the decellularization process. More importantly, through multi-level characterization, we comprehensively revealed the superiority of the TAS. Scanning electron microscopy (SEM) visually demonstrated the interconnected porous network structure inside the scaffold, providing a physical basis for cell adhesion, growth, and nutrient exchange. Proteomic analysis provided the strongest evidence for the scaffold’s bioactivity at the molecular level. Our LC-MS/MS results showed that various key structural and functional ECM proteins were effectively preserved, such as multiple types of collagen (COL1A1, COL3A1, COL4A1), laminin subunits (LAMA4, LAMB1, LAMC1), and fibronectin (FN1). These proteins collectively form the “skeleton” of the scaffold, not only providing mechanical support for cells but also exposing binding sites that are crucial for mediating cell adhesion, migration, and signal transduction (Stearns-Reider et al., 2023; Sun AR. et al., 2025). In particular, the retention of various proteoglycans such as HSPG2 (Perlecan), BGN (Biglycan), and DCN (Decorin) is vital for maintaining the homeostasis and function of the TME, as they can regulate the bioavailability of growth factors, participate in the assembly of collagen fibers, and directly influence cell behavior (Samaržija et al., 2024; Mongiat et al., 2024). In stark contrast, the vast majority of intracellular proteins, such as vimentin (VIM) and tubulin (TUBA1B), as well as intercellular adhesion molecules (VCAM1, ICAM1), were successfully removed. This result confirms that our decellularization method precisely removed potentially immunogenic cellular components while completely preserving the “biochemical fingerprint” that constitutes the tissue-specific microenvironment.

Furthermore, through ELISA quantitative detection, we found that the decellularized scaffold still retained a considerable amount of endogenous growth factors, including VEGF,TGF-β, HGF, FGF, EGF, and PDGF. These growth factors, naturally sequestered by the ECM, provide a continuous and physiological biological signal stimulus for the constructed 3D model (Zhang et al., 2021). Vascular endothelial growth factor (VEGF) is a key molecule regulating tumor angiogenesis (Apte et al., 2019); fibroblast growth factor (FGF) and platelet-derived growth factor (PDGF) are potent mitogens that can stimulate cell proliferation (Jung et al., 2025); and transforming growth factor-β (TGF-β) is a multifunctional cytokine that, in the later stages of tumor progression, acts as a key driver, strongly inducing epithelial-mesenchymal transition (EMT), promoting invasion, and mediating immune suppression (Shi et al., 2022; Chan et al., 2023). The retention of these growth factors in the scaffold, especially the presence of TGF-β, provides a direct molecular mechanism explanation for the EMT phenomenon (downregulation of E-cadherin, upregulation of N-cadherin and Vimentin) we observed in subsequent experiments with BCPAP cells. This endogenous, complex “cocktail” of growth factors, in synergy with the preserved ECM proteins, constitutes a signaling network that can authentically simulate the survival environment of thyroid cancer cells *in vivo*. At the same time, mechanical testing showed that the decellularization process did not significantly weaken the scaffold’s elastic modulus and ultimate tensile strength, and its mechanical properties remained in the same order of magnitude as native thyroid tissue. This high fidelity of biochemical signals and physical-mechanical cues is the core of the superiority of the 3D model constructed in this study.

Regarding cell proliferation, significant differences in proliferation kinetics were observed between the two culture methods, although both ultimately entered a plateau phase due to spatial or nutritional constraints. During the initial 10-day culture period, the 3D model demonstrated a significantly higher proliferation rate and total cell count compared to the 2D group. This enhanced viability was further substantiated by Live/Dead staining on day 15, which revealed a significantly greater proportion of viable cells within the 3D scaffold. Our findings on apoptosis provide a direct explanation for this pro-survival state. Flow cytometry analysis confirmed a significantly reduced rate of spontaneous apoptosis in the 3D TAS mode, indicating that the 3D microenvironment functions as an active, protective niche rather than a passive support. This reduction in baseline apoptosis likely contributes directly to the superior net proliferation observed. Collectively, these data indicate that the biomimetic microenvironment of the TAS, characterized by its 3D architecture, abundant adhesion sites, and bioactive molecules, robustly promotes cell growth and survival. Furthermore, the proliferation plateau observed in the 3D culture more accurately simulates the growth dynamics of solid tumors *in vivo*, which typically exhibit decelerated growth following an initial rapid expansion due to internal cell crowding and limited nutrient/oxygen diffusion (Barbosa et al., 2021; Zhu et al., 2023). In contrast, the 2D culture plateau is predominantly an artifact of contact inhibition upon reaching confluency, occurring at a substantially lower overall cell density. This demonstrates that our 3D model more faithfully recapitulates the macroscopic growth patterns of tumors.

At the microscopic level, the results from laser scanning confocal microscopy revealed the dynamic invasive behavior of the cells. Our results show that this invasion is a time-dependent process. On day 5 of culture, BCPAP cells were mainly enriched on the surface and in the shallow layers (10 μm) of the scaffold, with relatively low infiltration rates into deeper layers (50 μm and 100 μm), at 48.9% and 23.4%, respectively. However, by day 10, the cells’ infiltration ability was significantly enhanced, with the relative infiltration rates at 50 μm and 100 μm depths jumping to 71.3% and 42.7%, respectively (p < 0.01). This gradual infiltration from the surface to the interior is not a passive diffusion of cells but an active biological process. It indicates that tumor cells, in the biomimetic microenvironment provided by the TAS, can perceive and respond to ECM signals, activating their intrinsic migration and invasion programs (Hill et al., 2022). The preserved porous fibrous network of the scaffold provides physical channels for cell migration, while the rich ECM components (such as collagen and fibronectin) provide adhesion sites and “tracks” for cells to crawl on. More importantly, this functional enhancement of invasive ability is highly consistent with the molecular phenotype changes we observed. In the 3D environment, the cells underwent epithelial-mesenchymal transition (EMT)—downregulating E-cadherin and upregulating N-cadherin and Vimentin. EMT endows tumor cells with the ability to degrade the matrix, disrupt cell-cell junctions, and acquire high migratory capacity, which is precisely the functional manifestation of the enhanced infiltration phenomenon we observed on day 10 (Abuwatfa et al., 2024). Therefore, this infiltration process from the surface to the interior visually and quantitatively simulates the key early steps of metastasis, where tumor cells break through the basement membrane and invade the surrounding stroma, providing a highly valuable platform for studying the dynamic process and molecular mechanisms of tumor invasion (Nazari et al., 2023).

Particularly noteworthy is that the 3D culture environment profoundly reshaped the gene expression profile of thyroid cancer cells and induced their transformation towards a more aggressive malignant phenotype. Our qPCR results showed that, compared to the 2D group, the expression level of the BRAF V600E gene in the 3D model was significantly upregulated. As the core driving gene mutation in papillary thyroid carcinoma, the continuous activation of the BRAF V600E signaling pathway is key to tumorigenesis, development, and the maintenance of the malignant phenotype (Sun D. et al., 2025; Alnwisser et al., 2025). In traditional 2D culture, cells are detached from their original microenvironment, and their gene expression patterns may drift, failing to fully reflect their true state *in vivo*. Our findings suggest that the tissue-specific ECM microenvironment provided by the TAS may, through specific signaling pathways such as integrin-mediated mechanotransduction or signals from sequestered growth factors (like TGF-β), retroactively enhance the activity of the MAPK signaling pathway, thereby upregulating the expression of BRAF V600E (Fröhlich et al., 2023; Yuan et al., 2023). This indicates that our 3D model may more accurately reflect the functional state of this driver gene in real tumor tissue. Correspondingly, we observed a very clear phenomenon of Epithelial-Mesenchymal Transition (EMT). EMT is a key cellular biological process in which epithelial cells lose their polarity and tight cell-cell junctions, acquiring the morphology and migratory ability of mesenchymal cells. It is considered a core mechanism by which tumor cells acquire invasive and metastatic capabilities, generate stem cell properties, and induce drug resistance (Abuwatfa et al., 2024; Liaghat et al., 2024). In our 3D model, we observed a clear EMT phenomenon at the gene level. We have now confirmed this critical phenotypic switch at the protein level via immunofluorescence. Cells in the 3D model demonstrated a significant loss of E-cadherin and a concurrent gain of N-cadherin and Vimentin expression, both visually and quantitatively. This classic “cadherin switch” phenomenon strongly proves the initiation of the EMT process (Yang et al., 2020). This phenotypic transformation is likely driven by the complex signals within the TAS. For example, the TGF-β retained in the scaffold is one of the most potent known inducers of EMT (Chan et al., 2023); at the same time, the interaction of cells with ECM components (such as collagen and fibronectin) can activate various signaling pathways, including FAK, Src, and Rho/ROCK, all of which can promote cytoskeletal remodeling and the activation of EMT-related transcription factors (such as Snail, Slug, Twist) (Li et al., 2023; Tomecka et al., 2024). This finding is significant as it indicates that the natural ECM microenvironment provided by the TAS can effectively “push” tumor cells towards a more invasive and metastatic state, which is highly consistent with the process of tumor progression observed clinically.

Drug screening and drug resistance studies are among the most important application areas for *in vitro* tumor models and are a key criterion for judging their clinical predictive value. This study systematically evaluated the response of BCPAP cells in 2D and 3D models to two clinically common drugs—the broad-spectrum chemotherapy drug cisplatin and the BRAF V600E targeted drug Vemurafenib. The results clearly showed that, compared to cells that were extremely sensitive on a 2D plane, cells grown in the 3D scaffold exhibited significantly enhanced resistance to both drugs, with their half-maximal inhibitory concentration (IC50) values increasing by approximately 2.2-fold and 2.4-fold, respectively. This result profoundly reveals the major flaw of traditional 2D drug sensitivity tests—they systematically overestimate the efficacy of drugs, leading to the failure of many drugs that appear effective *in vitro* in clinical trials (Engelmann et al., 2020; Millen et al., 2023).

The drug resistance demonstrated by our 3D model can be attributed to multiple complex mechanisms that coexist in real tumors. First, the dense ECM fibrous network of the scaffold forms the first “physical barrier.” It can not only delay the penetration and diffusion of drug molecules through steric hindrance but may also non-specifically bind with drug molecules through its abundant functional groups, thereby reducing the effective drug concentration that reaches the tumor cells (Henke et al., 2020; Huang et al., 2021). Second, and more importantly, is “Cell Adhesion-Mediated Drug Resistance” (CAM-DR). Tumor cells bind to ligands in the ECM (such as fibronectin, collagen) through their surface integrin family receptors. This adhesion itself can activate a series of powerful pro-survival signaling pathways, such as the FAK/PI3K/Akt and Ras/MEK/ERK pathways (Jakubzig et al., 2018; Pang et al., 2023). These pathways can upregulate the expression of anti-apoptotic proteins (e.g., Bcl-2, Mcl-1) and downregulate the activity of pro-apoptotic proteins (e.g., Bax, Bad), thereby directly antagonizing the apoptosis program induced by chemotherapy drugs like cisplatin. For the targeted drug Vemurafenib, ECM-mediated signals may bypass the inhibited BRAF V600E target by activating parallel signaling pathways (e.g., activating other receptor tyrosine kinases, RTKs) or downstream pathway nodes, leading to adaptive resistance (Nocquet et al., 2024; Diazzi et al., 2023). Our new findings provide strong experimental support for this hypothesis. We demonstrated that the resistance to Vemurafenib in the 3D model is not permanent. By co-administering either a MEK inhibitor (Trametinib) or a FAK inhibitor (Defactinib), we successfully re-sensitized the 3D-cultured cells to Vemurafenib, resulting in significant enhanced cell death. This strongly suggests that the 3D microenvironment provided by TAS actively confers resistance by activating these parallel FAK and MEK signaling cascades, a mechanism that is completely absent in 2D culture. This highlights the critical importance of our 3D model as a platform for identifying and testing rational drug combinations designed to overcome resistance.

In addition, cells in the interior of the 3D culture may enter a slow-growing or dormant state (G0 phase) due to insufficient nutrient and oxygen supply. Since most chemotherapy drugs and some targeted drugs primarily act on rapidly proliferating cells, these quiescent cells are naturally insensitive to drugs and become seeds for tumor recurrence (Lv et al., 2016). At the same time, the 3D culture model is prone to forming a hypoxic microenvironment. We have now experimentally validated this hypothesis. Immunofluorescence staining confirmed a strong nuclear accumulation of HIF-1α in cells within the 3D scaffold, a phenomenon absent in 2D culture. This direct evidence of a hypoxic microenvironment is a key finding. The resulting induction of HIF-1α is known to further promote EMT, angiogenesis, and upregulate the expression of multi-drug efflux pumps (e.g., P-glycoprotein), exacerbating the drug resistance (Aggarwal et al., 2020; Chen Z. et al., 2023). Finally, as mentioned earlier, the EMT process induced by the 3D environment is itself an important drug resistance mechanism. Cells that have acquired a mesenchymal phenotype generally have stronger survival capabilities and resistance to apoptosis (Liaghat et al., 2024). In summary, our 3D model can integrate physical, biochemical, and cellular state factors to comprehensively reproduce the drug-resistant microenvironment of tumors. Therefore, it can serve as a more reliable and clinically predictive platform for screening truly effective anti-cancer drug combinations and for in-depth research into the molecular mechanisms of drug resistance.

Although this study successfully constructed and validated a functional 3D thyroid cancer model, demonstrating great application potential, we must also be aware of its limitations and use them as a basis for future research directions. First, the current model uses only a single, immortalized human thyroid cancer cell line, BCPAP. Although BCPAP is a classic tool for studying BRAF V600E mutation-positive thyroid cancer, it cannot represent the high heterogeneity of thyroid cancer, including different pathological subtypes (such as follicular carcinoma, anaplastic carcinoma) and individual differences between patients. To make the model more clinically representative, future research must aim to incorporate a wider variety of thyroid cancer cell lines, and more ideally, to use primary tumor cells isolated from patient surgical specimens (Patient-Derived Cells, PDCs) or to establish patient-derived tumor organoids (PDOs) (Chen D. et al., 2023; Yang et al., 2023). By seeding a patient’s own tumor cells onto the TAS, we hope to construct “personalized” tumor models (Avatar models) for screening postoperative adjuvant therapy regimens and predicting the efficacy of new drugs, thereby truly achieving precision medicine (Liu et al., 2023). Second, the current 3D model is a relatively simplified system. Although it includes the core ECM components and tumor cells, it still lacks other crucial cellular components of the TME. In a real tumor, tumor cells engage in complex and dynamic “dialogues” with cancer-associated fibroblasts (CAFs), various immune cells (such as tumor-associated macrophages, regulatory T cells), and vascular endothelial cells. These interactions profoundly affect tumor growth, invasion, angiogenesis, and immune evasion (Zhao et al., 2023; Wright et al., 2023). Therefore, an important future research direction will be to conduct multi-cell co-cultures on the TAS to construct more complex “organoid-like” models that can more completely simulate the TME ecosystem. Finally, this study utilized rat tissue to prepare the scaffold, as it is a standardized model suitable for our proof-of-concept research. However, we acknowledge the translational limitations of this xenogeneic approach. Species-specific differences in the ECM and potential immunogenicity could influence cell behavior and limit future *in vivo* applications. Therefore, our next step is to apply this validated protocol to human thyroid tissue. By using discarded surgical samples to create patient-specific models, we aim to significantly enhance the clinical relevance of our platform, moving closer to personalized medicine.

## 5 Conclusion

In conclusion, this study, through an optimized decellularization method, successfully prepared a thyroid acellular scaffold that retains the native tissue-specific structure and bioactivity. The 3D *in vitro* thyroid cancer model constructed based on this scaffold more closely simulates the biological characteristics of *in vivo* tumors than traditional 2D culture models in multiple dimensions, including cell proliferation kinetics, invasive behavior, key oncogene expression, EMT phenotype transformation, and drug response. The establishment of this model not only provides an unprecedented high-fidelity research platform for in-depth investigation into the pathogenesis, invasion and metastasis patterns, and drug resistance mechanisms of thyroid cancer, but also offers a more reliable and clinically predictive preclinical evaluation tool for developing new anti-cancer drugs, screening effective treatment regimens, and ultimately achieving personalized precision medicine. This work represents a solid and meaningful step forward in advancing the *in vitro* research paradigm for thyroid cancer and other solid tumors, moving from “flat” to “three-dimensional” and from “simplified” to “biomimetic.”

## Data Availability

The original contributions presented in the study are included in the article/supplementary material, further inquiries can be directed to the corresponding author.
